# Tuning the Size of Large Dense‐Core Vesicles and Quantal Neurotransmitter Release via Secretogranin II Liquid–Liquid Phase Separation

**DOI:** 10.1002/advs.202202263

**Published:** 2022-07-27

**Authors:** Zhaohan Lin, Yinglin Li, Yuqi Hang, Changhe Wang, Bing Liu, Jie Li, Lili Yin, Xiaohan Jiang, Xingyu Du, Zhongjun Qiao, Feipeng Zhu, Zhe Zhang, Quanfeng Zhang, Zhuan Zhou

**Affiliations:** ^1^ State Key Laboratory of Membrane Biology and Beijing Key Laboratory of Cardiometabolic Molecular Medicine Institute of Molecular Medicine College of Future Technology Peking‐Tsinghua Center for Life Sciences, and PKU‐IDG/McGovern Institute for Brain Research Peking University Beijing 100871 China

**Keywords:** adrenal chromaffin cells, large dense‐core vesicles, phase separation, quantal neurotransmitter release, secretogranin II, vesicle size

## Abstract

Large dense‐core vesicles (LDCVs) are larger in volume than synaptic vesicles, and are filled with multiple neuropeptides, hormones, and neurotransmitters that participate in various physiological processes. However, little is known about the mechanism determining the size of LDCVs. Here, it is reported that secretogranin II (SgII), a vesicle matrix protein, contributes to LDCV size regulation through its liquid–liquid phase separation in neuroendocrine cells. First, SgII undergoes pH‐dependent polymerization and the polymerized SgII forms phase droplets with Ca^2+^ in vitro and in vivo. Further, the Ca^2+^‐induced SgII droplets recruit reconstituted bio‐lipids, mimicking the LDCVs biogenesis. In addition, SgII knockdown leads to significant decrease of the quantal neurotransmitter release by affecting LDCV size, which is differently rescued by SgII truncations with different degrees of phase separation. In conclusion, it is shown that SgII is a unique intravesicular matrix protein undergoing liquid–liquid phase separation, and present novel insights into how SgII determines LDCV size and the quantal neurotransmitter release.

## Graphical abstract

The mystery of what fundamentally determines the size of large dense‐core vesicles (LDCVs) in neuroendocrine cells is uncovered. Secretogranin II, an intravesicular matrix protein, undergoes liquid–liquid phase separation in vitro and in vivo to determine the size/volume of LDCVs, thus tuning the quantal neurotransmitters release.

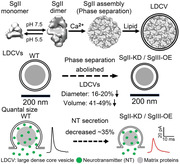

## 1 Introduction

In neurons and neuroendocrine cells, regulated secretion of signaling molecules occurs principally from two organelles, small synaptic vesicles (SSVs) and large dense‐core vesicles (LDCVs). SSVs (≈40 nm in diameter) are involved in the release of classical neurotransmitters only, while neuropeptides and neurotrophins are secreted via larger LDCVs (100–500 nm in diameter) with a characteristic electron‐dense core on transmission electron microscopy (EM).^[^ ^1^ ^]^ Neuropeptides are essential in regulating brain development, neurogenesis, and synaptic plasticity.^[^ ^2^, ^3^, ^4^ ^]^ Abnormal neuropeptide release has been associated with many diseases, such as cognitive deficits, psychiatric disorders, drug addiction, and obesity.^[^ ^3^, ^5^, ^6^, ^7^ ^]^ However, what fundamentally determines the size of LDCVs is still poorly understood.

In mammals, LDCVs are most abundant in peripheral neuroendocrine cells, especially the sympathetic neuroendocrine adrenal chromaffin cell (ACC), which is a classical neuroendocrine model for the study of quantal neurotransmitter release.^[^ ^8^, ^9^, ^10^ ^]^ Each ACC has ≈20 000 LDCVs and each LDCV mainly contains 0.8–1 M catecholamines,^[^ ^11^, ^12^ ^]^ ≈150 mM ATP,^[^ ^13^ ^]^ ≈40 mM Ca^2+^,^[^ ^14^ ^]^ and various neuropeptides, especially the intravesicular matrix proteins‐chromogranins.^[^ ^15^, ^16^, ^17^ ^]^ Chromogranins are the major component of the secretory granules or LDCVs in most neuroendocrine cells and they are involved in dense‐core granulogenesis, secretory protein cargo sorting, and as a source of bioactive peptides.^[^ ^18^, ^19^, ^20^ ^]^

The chromogranin family mainly includes chromogranin A (CgA),^[^ ^21^ ^]^ chromogranin B (CgB),^[^ ^22^ ^]^ and secretogranin II (SgII, or CgC).^[^ ^23^ ^]^ CgA and CgB account for ≈90% of the total intravesicular matrix proteins in ACC,^[^ ^24^ ^]^ and they have been intensively studied to bind to and enrich high concentrations of catecholamines and Ca^2+^ in the LDCVs.^[^ ^12^, ^25^, ^26^, ^27^, ^28^, ^29^ ^]^ CgA or CgB knockout or CgA/B double knockout produces a similar 30–40% reduction of quantal catecholamine release from LDCVs, but their effects on the size of LDCVs are controversial.^[^ ^12^, ^26^, ^28^, ^30^, ^31^, ^32^, ^33^, ^34^, ^35^ ^]^ SgII accounts for only ≈1% of total intravesicular matrix proteins in LDCVs and is known to bind to Ca^2+^ with high capacity and low affinity in vitro.^[^ ^36^, ^37^ ^]^ Nonetheless, whether SgII plays a role in regulating LDCVs morphology or function remains unknown in native neuroendocrine cells.

The amount of neurotransmitter release from individual vesicles triggered by Ca^2+^ influx is defined as quantal size (QS), which determines the strength and speed of synaptic transmission.^[^ ^38^, ^39^ ^]^ The electrochemical carbon fiber electrode (CFE) provides a fast and exquisitely sensitive tool for real‐time, in situ monitoring of single LDCV catecholamine quantal release by electro‐oxidation in ACCs.^[^ ^26^, ^40^, ^41^ ^]^ It is known that QS of LDCVs can be regulated by proteins/ions outside the vesicles via affecting vesicle fusion pore dilation; these include the G protein‐coupled receptor *βγ*, the SNARE complex, dynamin, and Ca^2+^.^[^ ^42^, ^43^, ^44^, ^45^, ^46^, ^47^ ^]^ Also, the change in vesicle size/volume of LDCVs is associated with the corresponding change in QS.^[^ ^48^, ^49^, ^50^ ^]^ Lately, we revealed that CgA is an essential factor for the formation of sub‐quantal release via binding to catecholamines and limiting their diffusion speed from a small vesicle fusion pore,^[^ ^26^ ^]^ suggesting critical roles of intravesicular matrix proteins in LDCV quantal secretion. This discovery led us to pay more attention to the internal proteins‐chromogranins (including SgII) and their effect on LDCV release.

Recently, many components in cells have been verified to undergo liquid–liquid phase separation to form granules by multivalent interactions through intrinsically disordered regions or binding partners.^[^ ^51^, ^52^ ^]^ The phase separation assembly, formed by concentrated molecules that stably exist in a liquid milieu, is a kind of membrane‐less compartment (also called biomolecular condensate) and has been found in nucleoli, cytosol, and organelles.^[^ ^51^, ^53^, ^54^, ^55^ ^]^ Several studies have revealed that protein phase separation plays a pivotal role in many physiological processes, including autophagic degradation, gene expression, regulation of enzyme activity, formation of the skin barrier, vesicle clustering, and formation of postsynaptic densities.^[^ ^56^, ^57^, ^58^, ^59^, ^60^, ^61^, ^62^ ^]^ However, many granule‐liked structures (such as LDCVs in neuroendocrine cells) are still not fully understood, and whether these structures or LDCVs are formed by phase separation is unknown.

Here, by combining electrochemical CFE recording (detection of single LDCV catecholamine release), biochemistry (protein purification), structural biology (cryo‐electron microscopic analysis of single particles and correlative light‐EM (CLEM)), and confocal imaging (phase separation), we demonstrated that SgII plays a vital role in regulating the size of LDCVs via liquid–liquid phase separation, which further determines the quantal neurotransmitter release in native ACCs.

## 2 Results

### 2.1 QS is Decreased in SgII Knockdown (SgII‐KD) ACCs

**Figure 1:**
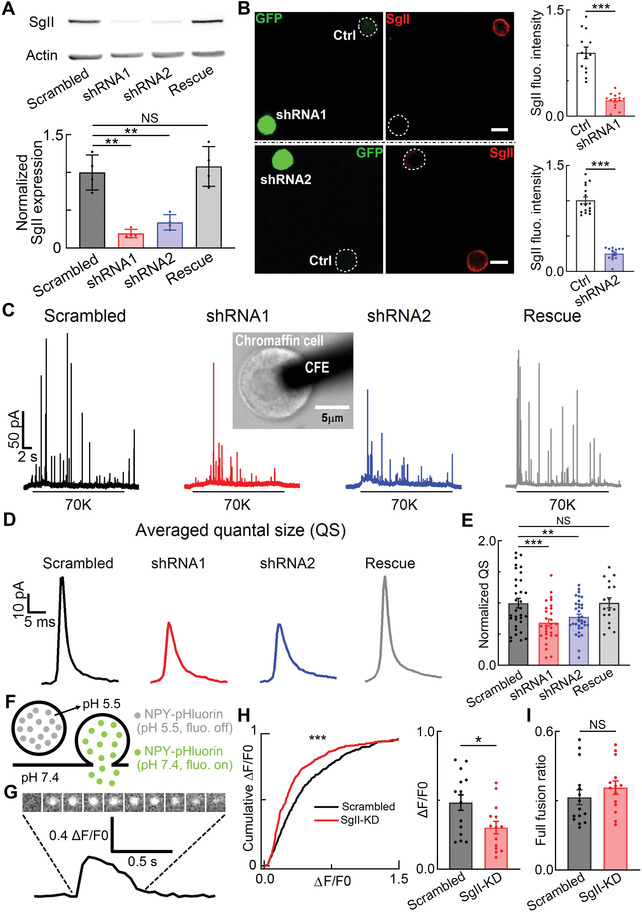
SgII‐KD reduces quantal release of LDCVs in ACCs. A) Western blots and statistics of SgII expression in Cos‐7 cells transfected with scrambled, SgII‐KD‐shRNA1, SgII‐KD‐shRNA2, or shRNA1‐resistant SgII‐rescue plasmids (*n* = 4 independent experiments, one‐way ANOVA). SgII expression is efficiently knocked down and rescued by two shRNAs and SgII‐rescue plasmids. B) Representative micrographs of immunofluorescent staining and statistics of SgII expression in SgII KD (shRNA1, upper panels; shRNA2, lower panels) chromaffin cells (*n* = 13 ctrl and 14 shRNA1 cells; *n* = 15 ctrl and 13 shRNA2 cells, unpaired Student's *t*‐test). Scale bars, 10 µm. C) Representative amperometric recordings of quantal catecholamine release in response to a 20‐s stimulus of 70 mM KCl (70 K) in scrambled, SgII‐KD‐shRNA1, SgII‐KD‐shRNA2, and SgII‐rescue ACCs (inset micrograph of a CFE (black bar) attached to an ACC membrane during amperometric recording). D) Averaged QS in scrambled, SgII‐KD‐shRNA1, SgII‐KD‐shRNA2, and SgII‐rescue ACCs. E) Statistics of normalized QS corresponding to panel (D) (*n* = 33 for scrambled cells, 29 for shRNA1 cells, 34 for shRNA2 cells, and 17 for rescued cells; one‐way ANOVA). F) Cartoon of two NPY‐pHluorin fluorescent states at pH 5.5 (vesicular environment, fluorescence off) and pH 7.4 (extracellular environment, fluorescence on). G) Upper, representative images of single LDCV release from NPY‐pHluorin transfected ACC under TIRF imaging. Image size, 1 µm × 1 µm; 53 ms per frame. Lower, the normalized fluorescent intensity (Δ*F*/*F*0) curve, corresponding to upper images. More details seen in Figure S1F,G, Supporting Information. H) Compare to scrambled ACCs, the cumulative Δ*F*/*F*0 (left) and average Δ*F*/*F*0 (right) both decrease in SgII‐KD ACCs (total 378 fusion events from *n* = 15 scrambled cells and total 324 fusion events from *n* = 14 SgII‐KD cells; K–S test and unpaired Student's *t*‐test). I) The full fusion ratio in scrambled and SgII‐KD ACCs made no difference (unpaired Student's *t*‐test). Data are presented as the mean ± SEM with scatter dots (A, B, E, H, I). *P < 0.05, **P < 0.01, ***P < 0.001; NS, no significant difference.

CgA, CgB, and SgII are the main matrix proteins in LDCVs.^[^ ^18^ ^]^ Since CgA and CgB have been demonstrated to affect catecholamine release in ACCs,^[^ ^26^, ^34^ ^]^ we next tested whether SgII regulates quantal catecholamine release. First, we designed two small hairpin RNA interference (shRNAi) molecules to knock down SgII expression in mouse ACCs. Compared with scrambled (control) cells, Western blot analysis of Cos‐7 cells (SgII overexpression forms LDCVs in Cos‐7 cells^[^ ^63^ ^]^) and immunofluorescent staining of ACCs revealed ≈70% reduction of SgII in SgII‐KD cells by the two shRNAs, and the reduction of SgII could be rescued by an shRNA‐1‐resistant form of SgII (**Figure** **1**A,B, and Figure S1A,B, Supporting Information). Hence, we were convinced that our shRNAs successfully target SgII in ACCs. Next, by using CFEs to directly touch the membrane surface of ACCs, we recorded a burst of oxidized amperometric spikes that was evoked by high KCl (70 mM) to depolarize the ACCs. Each spike signal represents quantal catecholamine release from single LDCV, named QS (Figure 1C, see also refs. [26, 44]). The average QS was significantly reduced by 35% in SgII‐KD ACCs by the two shRNAs, and this reduction could be fully rescued by shRNA‐1‐resistant SgII (Figure 1C–E and Figure S1C, Supporting Information). We further thoroughly analyzed the other kinetic parameters of the amperometric spikes (Figure S1D, Supporting Information) and found that the peak amplitude was also decreased by ≈30%, while the half‐height duration and rise time (RT) remained unchanged (Figure S1E, Supporting Information). The CFE data showed that the depletion of SgII in ACCs led to an obvious reduction of quantal catecholamine release. Besides, we wondered whether the neuropeptides in LDCVs were also influenced by SgII‐KD. Neuropeptide Y (NPY) is native in ACC,^[^ ^17^, ^64^ ^]^ making NPY‐pHluorin an ideal indicator to visualize the single LDCV release under total internal reflection fluorescence (TIRF) microscopy (Figure 1F).^[^ ^26^, ^45^, ^65^, ^66^ ^]^ Compared to scrambled ACCs, quantal NPY release in SgII‐KD ACCs exhibited smaller ΔF/F0 (Figure 1G,H, Figure S1F,H and Movies S1 and S2, Supporting Information), which directly reflected the cargo content in SgII‐KD cells were diminished. While the full fusion ratio made no difference in these two groups (Figure 1I and Figure S1G, Supporting Information). In summary, these results demonstrate that the intravesicular matrix protein SgII regulates quantal release of LDCVs in native ACCs.

### 2.2 SgII Regulates QS by Controlling LDCV Size

**Figure 2:**
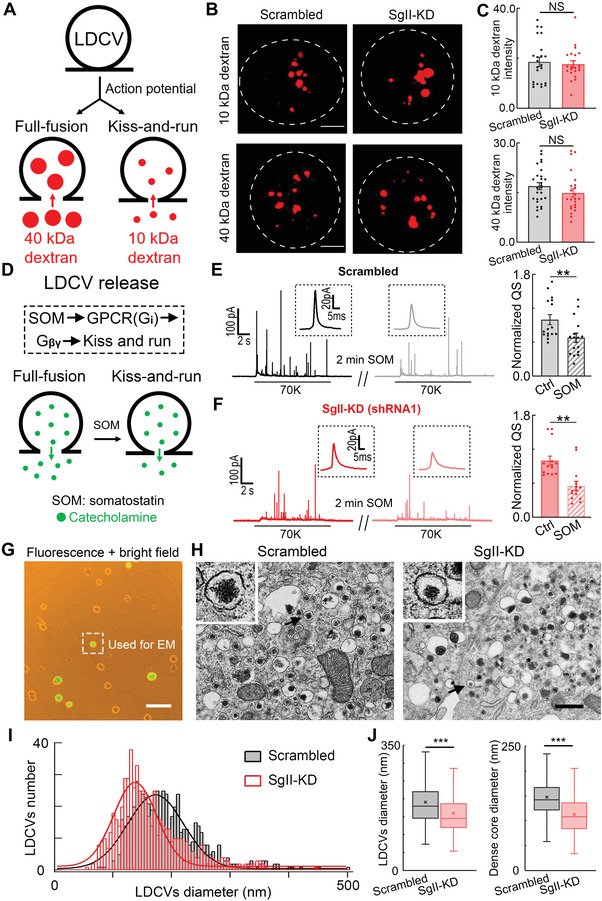
SgII regulates LDCV size by using CLEM, but not the fusion pore in ACCs. A) Cartoon of TRM‐dextran uptake through open fusion pores during action potential‐induced LDCV exocytosis. Both fusion modes (“full‐fusion” and “kiss‐and‐run”) can take up small dextran nanoparticles (10 kDa, diameter Φ 5 nm), but full fusion mode can take up more large dextran nanoparticles (40 kDa, diameter Φ 9 nm) in ACCs (see also refs. [26, 45]). B) Representative z‐projection confocal images show the uptake of 10 and 40 kDa TMR‐dextran nanoparticles in scrambled and SgII‐KD ACCs during 70 mM KCl stimulation for 2 min. C) Statistics of 10 and 40 kDa TMR‐dextran uptake intensity in scrambled and SgII‐KD ACCs (for 10 kDa: *n* = 23 scrambled and 21 SgII‐KD cells; for 40 kDa: *n* = 27 scrambled and 23 SgII‐KD cells; unpaired Student's *t*‐test). D) Cartoon showing two release modes of LDCVs in ACCs: full‐fusion (quantal or complete release) and kiss‐and‐run (sub‐quantal or partial release). Somatostatin (SOM, a GPCR‐Gi activator) is used to restrict fusion pore expansion and switch the mode from full‐fusion to kiss‐and‐run.^[^ ^26^ ^]^ E) Left, representative amperometric recordings before and after 2 min SOM (500 nM) treatment in scrambled ACCs. Right, quantification of QS reduction by SOM (*n* = 16, paired Student's *t*‐test). F) Similar to (E) but in SgII‐KD (shRNA1) ACCs (*n* = 14, paired Student's *t*‐test). G) CLEM to precisely locate the scrambled and SgII‐KD ACCs. Bright and fluorescence fields show two kinds of ACCs transfected (green fluorescence) or not transfected (no fluorescence) with the target plasmids (scrambled or SgII‐KD). Cell location is spotted in bright‐field and successfully transfected cells can be selected for further EM experiments. Scale bar, 200 µm. H) Representative transmission EM micrographs of LDCVs from scrambled and SgII‐KD ACCs. Scale bar, 500 nm. I) Distribution of LDCV diameters showing smaller vesicles in SgII‐KD cells (pink) than scrambled cells (gray). J) Histograms show that SgII‐KD leads to a reduction of LDCV diameter by ≈16% and dense core diameter by ≈23%. The statistics of I,J) are from 601 LDCVs for scrambled cells and 597 LDCVs for SgII‐KD cells (K–S test). Data are presented as the mean ± SEM with scatter dots (C,E,F) or the medians with interquartile ranges (J). **P < 0.01, ***P < 0.001; NS, no significant difference.

Next, we explored the mechanism underlying the regulation of QS by SgII. It is known that the QS of LDCVs is regulated by several key factors, including the vesicle fusion mode (full‐fusion versus kiss‐and‐run), transmitter binding affinity with intravesicular matrix proteins, and vesicle size.^[^ ^26^, ^34^, ^50^ ^]^ In native ACCs, LDCVs undergo exocytosis in two fusion modes: “full‐fusion” with a larger fusion pore and “kiss‐and‐run” with a smaller fusion pore (**Figure** **2**A, and see also ref. [45]). To address whether the fusion pore becomes smaller after SgII‐KD, we used the fact that an exocytic LDCV captures or excludes external probes of different sizes based on the extent of fusion pore dilation before re‐closure.^[^ ^26^, ^45^, ^67^ ^]^ We found that the fluorescent nanoparticles dextran 10 kDa (Φ 5 nm) and 40 kDa (Φ 9 nm) were both captured by LDCVs with similar intensity in scrambled and SgII‐KD ACCs (Figure 2B,C), suggesting that the fusion pore size does not differ between these two groups. This result was consistent with the TIRF imaging result that the full‐fusion ratio (full‐fusion events number/total events number) made no difference in scrambled and SgII‐KD ACCs (Figure 1I), indicating that the LDCV fusion mode was not altered by SgII‐KD. Furthermore, somatostatin (SOM, a GPCR‐Gi activator) was applied to ACCs during vesicle exocytosis to restrict fusion pore expansion and switch the fusion mode from full‐fusion to kiss‐and‐run (Figure 2D).^[^ ^26^, ^44^ ^]^ We found that similar to scrambled cells, SOM still reduced QS in SgII‐KD cells (Figure 2E,F and Figure S2A–D, Supporting Information), demonstrating that the fusion pore contraction of LDCVs (expanding and shrinking) was normal in SgII‐KD ACCs. Then, the binding affinity between SgII and a representative catecholamine, norepinephrine (NE), was measured by surface plasma resonance (SPR) analysis. Their dissociation constants (*K*_d_) were 249 mM at pH 7.5 and 284 mM at pH 5.5 (Figure S2E,F, Supporting Information). Considering that the *K*_d_ between whole chromogranins and NE is 2.1 mM and one major chromogranin, CgA, binds to fivefold NE molecules,^[^ ^25^, ^29^ ^]^ we suggest that the weak ≈250 mM binding affinity between SgII and NE does not contribute to large NE storage in LDCVs, which excludes the possibility of catecholamine deficiency in SgII‐KD ACCs.

Furthermore, CLEM was applied to seek morphological differences between scrambled and SgII‐KD ACCs. CLEM precisely located scrambled and SgII‐KD cells to ensure that every analyzed cell was successfully transfected with the corresponding plasmid (Figure 2G). Quantification of ≈600 LDCVs in each group, showed that the maximum distributions of LDCV diameter were 150–200 nm in scrambled and 100–150 nm in SgII‐KD cells (Figure 2H,I). The average diameters of LDCVs and dense cores in SgII‐KD cells were smaller than those in scrambled cells by ≈16% and ≈23%, respectively, so the volume of LDCVs decreased by nearly 40% (Figure 2J and Figure S2G,H, Supporting Information). These data together suggest that SgII modulates QS by affecting the size of LDCVs in ACCs.

### 2.3 Polymerization of SgII is pH‐Dependent

**Figure 3:**
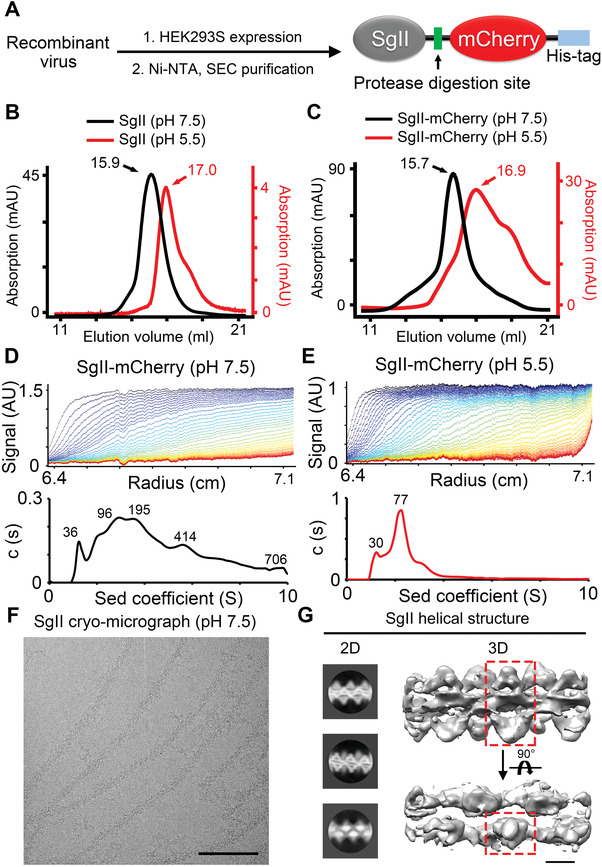
SgII shows pH‐dependent polymerization. A) Flowchart of protein expression and purification. Proteins are expressed in the Bac‐Mam system, and a His‐mCherry tag is attached to the C‐terminal of SgII. B) Size exclusion chromatography (SEC) profiles of SgII purification at pH 7.5 (black) and pH 5.5 (red). The elution volume of the peak fraction is 15.9 mL for pH 7.5 and 17.0 mL for pH 5.5, indicating that SgII has more polymerization at pH 7.5 than at pH 5.5. C) SEC profiles of SgII‐mCherry purification at pH 7.5 (black) and pH 5.5 (red). The elution volumes of the peak fraction are pointed and labeled, as in panel (B). D,E) AUC studies of SgII‐mCherry at pH 7.5 (left) and pH 5.5 (right). Upper panels, absorbance at 280 nm (vertical axis) versus time (horizontal axis). Lower panels, molar mass distribution, c (s). Compared with pH 5.5 (E), SgII at pH 7.5 (D) exhibits marked polymerization. F) Representative cryo‐electron micrograph of SgII filaments at pH 7.5. Scale bar, 100 nm. G) 2D and 3D results for SgII filaments. Left panel, three typical orientations of the SgII helical structure projection in 2D classification. Right panel, two side‐views of the SgII 3D model. One dimeric subunit is outlined by red dashed lines. Scale bar, 50 Å.

How does SgII determine the size of LDCVs? Next, we explored the biochemical properties of SgII under both neutral (pH 7.5) and acidic (pH 5.5) conditions to mimic the immaturely and maturely vesicular environments.^[^ ^68^ ^]^ By introducing biochemical methods, SgII was overexpressed in HEK293S cells and then purified at different pH values (**Figure** **3**A,B and Figure S3A, Supporting Information). While, during purification, we found that the solubility and stability of SgII were much worse at the acidic pH (Figure 3B and Figure S3A, Supporting Information). Notably, we found that SgII tended to form higher oligomers under the neutral circumstance (pH 7.5), as evident by the left‐shift of elution volume on a Superose6 10/300 gel filtration column (15.9 mL at pH 7.5 versus 17.0 mL at pH 5.5) (Figure 3B). To verify that the oligomerization state of SgII does vary with a change in pH, we applied analytical ultracentrifugation (AUC) to accurately analyze its molecular weight. In this assay, we used mCherry‐tagged SgII (SgII‐mCherry) instead for its better stability at pH 5.5, and the gel filtration profiles showed that SgII‐mCherry had the same pH‐dependent oligomerization pattern, suggesting that the mCherry tag did not influence the biochemical properties of SgII (Figure 3C). The time‐serial sedimentation curves calculated from the AUC assays showed that SgII‐mCherry indeed was distributed in several high‐molecular‐weight fractions at pH 7.5, while it only appeared in the monomeric fraction at pH 5.5 (Figure 3D,E). Furthermore, we subjected the purified SgII protein at different pH values to cryo‐EM. Surprisingly, we found that SgII assembled into filaments under neutral but not acidic conditions (Figure 3F and Figure S3B, Supporting Information). 2D classification of 228 744 particles revealed that the filament structure had two‐fold helical symmetry, which was refined to 12.0° in azimuthal angle and 100.3 Å in axial rise per subunit in the final 3D model (Figure 3G and Figure S3C, Supporting Information). It seemed that each subunit is a dimeric SgII based on the shape and size of the cryo‐EM map (Figure 3G and Figure S3D, Supporting Information). The appearance of long helical filament is further strong evidence that SgII undergoes characteristic aggregation at pH 7.5. In short, our in vitro studies together demonstrated that SgII is inclined to aggregate and assemble into high polymers at pH 7.5, whereas it exists as monomer at pH 5.5.

### 2.4 Polymerized SgII Undergoes Liquid–Liquid Phase Separation with Proper Ca^2+^ Concentration In Vitro and In Vivo

**Figure 4:**
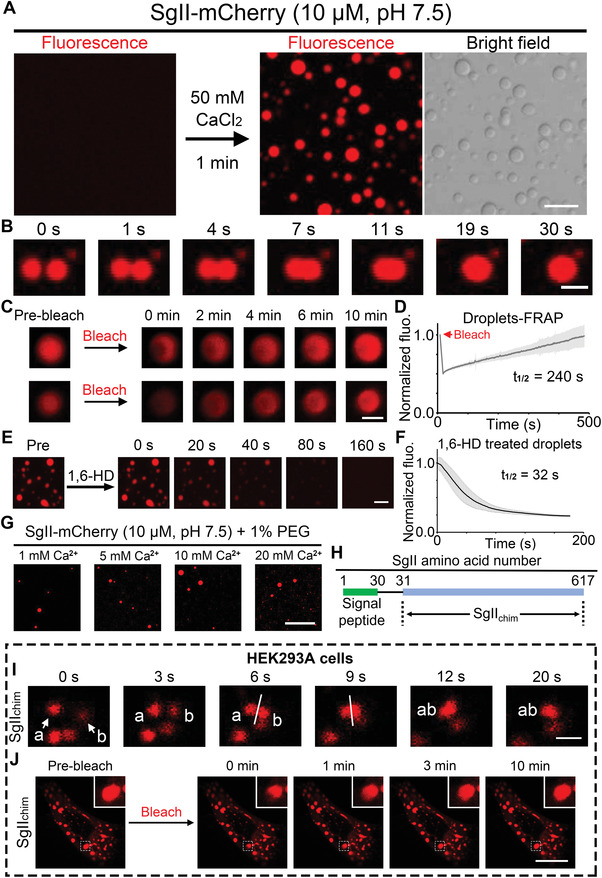
Ca^2+^ induces polymerized SgII to form liquid–liquid phase separation in vitro and in vivo. A) SgII‐mCherry (10 µM, pH 7.5) forms liquid droplets within minutes in the presence of 50 mM Ca^2+^ at 25 °C. Scale bar, 10 µm. B) Droplets of SgII show liquid behavior by fusing with each other and relaxing to round, bigger droplets within 30 s to minimize surface tension. Scale bar, 3 µm. C) FRAP: photobleaching of partial (upper panel) or full (lower panel) region of SgII droplets with subsequent recovery of fluorescence within 10 min. Scale bar, 2 µm. D) FRAP curve of SgII droplets from photobleaching recovery (*n* = 23 droplets). E) SgII droplets are dissipated within 2 min by adding 5% 1,6‐hexanediol (1,6‐HD). Scale bar, 10 µm. F) Statistics of fluorescence intensity of SgII droplets dissipated by adding 5% 1,6‐HD (*n* = 10 droplets). G) A crowding reagent (1% PEG) facilitates SgII to form droplets at the relatively lower Ca^2+^ concentrations ranging from 1 to 20 mM. Scale bar, 40 µm. H) SgII_chim(31–617)_: signal peptide (containing amino‐acids 1–30 of SgII) is removed from full‐length SgII (total of 617 amino‐acids). I) In HEK293A cells, SgII_chim_ granular structures fuse with each other and relax to a bigger granular structure within 20 s. Scale bar, 2 µm. J) FRAP in HEK293A cells: fluorescence intensity of partially photobleached SgII_chim_ granular structure recovers within 10 min. Scale bar, 15 µm.

Since SgII forms polymers at pH 7.5 but monomers at pH 5.5, we investigated whether the polymerized SgII can perform unique actions that monomers cannot. SgII binds to Ca^2+^ with high capacity,^[^ ^37^ ^]^ and Ca^2+^ is the most abundant ion in the organelles associated with secretory pathways, such as the endoplasmic reticulum (ER, 1–3 mM Ca^2+^,^[^ ^69^ ^]^) the Golgi complex (2.5‐fold of ER Ca^2+[^ ^70^ ^]^), and LDCVs (≈40 mM Ca^2+[^ ^14^ ^]^), so we first explored the effect of Ca^2+^ on SgII. We incubated 10 µM SgII‐mCherry with 50 mM CaCl_2_ at pH 7.5, and multiple micrometer‐sized spherical droplets appeared within 1 min and fused with each other dynamically as seen with fluorescent confocal living imaging (**Figure** **4**A,B and Movie S3, Supporting Information). Meanwhile, droplets also appeared when the same experiments were carried out with pure SgII (without the mCherry tag) and enhanced green fluorescent protein (EGFP)‐tagged SgII (Figure S4A, Supporting Information), strongly suggesting that the droplets were formed by SgII itself but not the tagged proteins. Next, to test whether the SgII droplets were organized by liquid–liquid phase separation, Fluorescence recovery after photobleaching (FRAP) assays were performed. FRAP is used to verify that the molecular constituents of liquid‐like droplets are indeed mobile.^[^ ^54^, ^71^ ^]^ The FRAP results showed that the partially or fully photobleached areas of droplets recovered fluorescence within 10 min after bleaching (Figure 4C,D and Movie S4, Supporting Information). These results conveyed to us that SgII in droplets had liquid phase properties: fusion, inside rearrangement, and in‐and‐out exchange. Then, we used 5% 1,6‐hexanediol (1,6‐HD), a widely used reagent to interfere with the phase separation,^[^ ^72^, ^73^ ^]^ to incubate with SgII phase droplets. We found that SgII‐mCherry droplets dissolved in 1–2 min after treatment with 1,6‐HD (Figure 4E,F and Movie S5, Supporting Information), reconfirming that the in vitro SgII phase separation was authentic.^[^ ^74^ ^]^ However, at pH 5.5, SgII did not form phase droplets even at higher protein and Ca^2+^ concentrations (Figure S4B, Supporting Information). Hence, we conclude that the SgII phase separation property relies on its polymerization at pH 7.5, but not the monomeric state at pH 5.5. Also, SgII phase separation had a divalent cation preference because we found that only the divalent cations Ca^2+^ and Mg^2+^ induced SgII droplet generation, whereas the monovalent cations Na^+^ and K^+^ did not (Figure S4C, Supporting Information). Moreover, the phase separation phenomenon was dose‐dependent on SgII and Ca^2+^ (Figure S4D,E, Supporting Information). Notably, 20 mM Ca^2+^ was the minimum concentration to induce SgII droplet formation in vitro at pH 7.5; this condition is higher than common Ca^2+^ concentration in most organelles. To mimic the crowded cellular environment in vivo, we added the crowding reagent polyethylene glycol 8000 (PEG)^[^ ^59^ ^]^ to the SgII buffer solution. 3% PEG (w/v) directly induced the formation of SgII droplets without adding Ca^2+^ (Figure S4F, Supporting Information), indicating that PEG indeed promoted SgII phase separation. To provide a more moderate circumstance, we also tested 1% PEG effect on the formation of SgII droplets, which may be more closed to the physiological state. In the presence of 1% PEG, lower Ca^2+^ concentrations (from 1 to 20 mM) also induce SgII droplet formation, and this phenomenon exhibited Ca^2+^ concentration dependence (Figure 4G). Also, FRAP results confirmed the dynamic liquid‐like character of SgII droplets in 1% PEG at 1 mM Ca^2+^ concentration (Figure S4G, Supporting Information). As controls, neither SgII at pH 5.5 nor mCherry‐tag alone at pH 7.5 formed droplets in 1% PEG buffer (Figure S4H, Supporting Information).

These in vitro studies suggest to us that SgII phase separation can happen in cells. Because the SgII signal peptide (SP, 1–30 amino‐acids of SgII) sorts SgII into vesicles (pH 5.5), to sort SgII in the cytosol and test whether SgII can form phase droplets in the neutral environment (pH 7.5), we constructed an SP‐deleted SgII plasmid tagged with mCherry (SgII_31–617_‐mCherry) (Figure 4H). We indeed observed multiple red granular structures in HEK293A cells after transfection with SgII_31–617_‐mCherry and these granules fused with each other, strongly suggesting that SgII can form phase separation in cells (Figure 4I). Meanwhile, the results of FRAP assays showed that the fluorescence intensity of partially or fully photobleached granular structures both recovered in 10–30 min (Figure 4J, Figure S4I,J and Movie S6, Supporting Information), again confirming that SgII granular structures at the cellular level were generated by phase separation and the granular proteins showed mobile liquid‐like characters. Taken together, we found that polymerized SgII formed liquid droplets via liquid–liquid phase separation with Ca^2+^ in both buffer solutions (in vitro, Figure 4A–G) and cells (in vivo, Figure 4H–J), which strongly indicated that the SgII phase separation phenomenon can be carried out in the organelles associated with vesicle synthesis and secretion in ACCs.

### 2.5 SgII Regulates LDCV Size and QS via Phase Separation in Native ACCs

**Figure 5:**
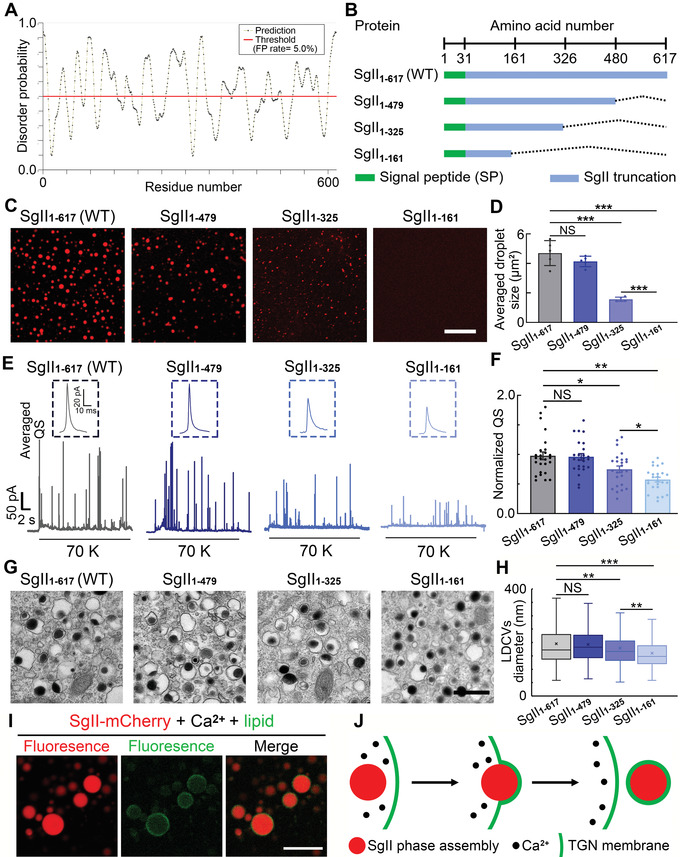
SgII regulates LDCV size and QS via phase separation in native chromaffin cells. A) Intrinsic disorder probability of SgII residues predicted by PrDOS (Protein DisOrder prediction System, https://prdos.hgc.jp/) (y‐axis, disorder probability; x‐axis, amino‐acid position). B) Design of SgII truncations. Wild‐type (WT, SgII_1–617_) and truncations with three‐quarters of the full‐length (SgII_1–479_), half of the full‐length (SgII_1–325_), and a quarter of the full‐length (SgII_1–161_) are constructed and used for subsequent experiments. C) Phase separation test for SgII truncations. Typical fluorescent micrographs of mCherry‐tagged SgII truncations with 50 mM Ca^2+^ (scale bar, 20 µm). D) Statistics of averaged phase droplet size of mCherry‐tagged SgII truncations corresponding to panel (C) (droplets are from 5 fluorescence micrographs per group, one‐way ANOVA). E) Representative amperometric recordings of quantal catecholamine release in response to a 20‐s stimulus of 70 mM KCl (70 K) in SgII_1–617_, SgII_1–479_, SgII_1–325_, and SgII_1–161_ rescued ACCs. Insets, averaged QS corresponding to SgII_1–617_, SgII_1–479_, SgII_1–325_, and SgII_1–161_ rescued ACCs. F) Statistics of normalized QS corresponding to panel (E) (*n* = 26 for SgII_1–617_ rescued ACCs, 25 for SgII_1–479_ rescued ACCs, 25 for SgII_1–325_ rescued ACCs, and 25 for SgII_1–161_ rescued ACCs; one‐way ANOVA). G) Representative transmission EM micrographs of LDCVs from SgII_1–617_, SgII_1–479_, SgII_1–325_, and SgII_1–161_ ACCs (scale bar, 500 nm). H) Statistics of LDCV diameters corresponding to panel (H) (*n* = 609 LDCVs for SgII_1–617_ rescued cells, 774 for SgII_1–479_ rescued cells, 956 for SgII_1–325_ rescued cells, and 766 for SgII_1–161_ rescued cells; K–S test). I) SgII‐mCherry phase droplets (SgII‐mCherry: 10 µM; Ca^2+^: 50 mM) can be coated by reconstituted bio‐lipids under confocal imaging (scale bar, 20 µm). More details see Figure S7A–G, Supporting Information. J) Cartoon shows how SgII phase droplets contribute to LDCV size. SgII phase droplets first bud at TGN membrane and then package into immature LDCV, transporting to cytoplasm. Data are presented as the mean ± SEM with scatter dots (D,F) or the medians with interquartile ranges (H). *P < 0.05, **P < 0.01, ***P < 0.001; NS, no significant difference.

A high content of positively charged (K, R, and H) and negatively charged (D and E) amino‐acid residues are even distributed in SgII (Figure S5A, Supporting Information) and the SgII intrinsic disordered regions predicted by PrDOS (Protein DisOrder prediction System) are also widely distributed (**Figure** **5**A). To identify which region or part is most essential for SgII phase separation, we constructed and purified several SgII truncations by quartering SgII: 1–617 (WT), 1–479 (3/4 WT), 1–325 (1/2 WT), and 1–161 (1/4 WT) tagged with mCherry (Figure 5B and Figure S5B, Supporting Information). The results of in vitro phase separation showed that SgII_1–479_ formed phase droplets comparable to SgII_1–617_ (WT), SgII_1–325_ formed smaller phase droplets, and SgII_1–161_ did not form any droplets under confocal imaging (Figure 5C,D). Several other SgII truncations were also tested for phase separation ability (Figure S5B,C, Supporting Information) and the truncations with three‐quarter length (SgII_1–325, 480–617_; SgII_1–161, 326–617_; and SgII_1–30, 162–617_) and one‐half length (SgII_1–30, 326–617_) had greatly weakened phase separation, and one quarter length truncations (SgII_1–30, 162–325_; SgII_1–30, 326–479_; and SgII_1–30, 480–617_) completely lost phase separation ability (Figure S5C,D, Supporting Information). Taken together, through systematically screening the SgII truncations, we have uncovered that the ability of SgII phase separation is related to the length of SgII, and the first three‐quarters of sequence are more important for phase separation.

Since liquid–liquid phase separation is formed by concentrated molecules and always shows dense structures under the electron microscope, such as a postsynaptic density domain^[^ ^61^ ^]^ and a skin barrier formed by keratohyalin granules,^[^ ^56^ ^]^ we hypothesized that the formation of dense‐core granules in ACC LDCVs is related to phase separation of the intravesicular matrix protein SgII. To answer this question, we overexpressed four typical SgII truncations (Figure 5B) into SgII‐KD ACCs to test their rescue effect on quantal catecholamine release (Figure 5E,F). We found that SgII_1–617_ (WT) fully rescued the QS of catecholamine release in SgII‐KD ACCs (Figures 5E and 1C–E). The QS rescued by SgII_1–479_ was comparable to that of SgII_1–617_ (WT), while the QS rescued by SgII_1–325_ or SgII_1–161_ was gradually decreased compared with that of SgII_1–617_ (WT) (Figure 5E,F and Figure S6A, Supporting Information). So SgII_1–479_ with complete phase separation fully rescued quantal catecholamine release, SgII_1–325_ with reduced phase separation only partially rescued the QS, and SgII_1–161_ without phase separation completely lost its rescue effect on QS in SgII‐KD ACCs (Figure 5C–F), strongly indicating that SgII regulates catecholamine QS via phase separation in ACCs. Also, by using CLEM, we found that the LDCVs and dense core diameters in ACCs rescued by SgII_1–479_ were comparable to those of ACCs rescued by WT, but became smaller in ACCs rescued by SgII_1–325_, and were smallest in ACCs rescued by SgII_1–161_ (Figure 5G,H and Figure S6B,C, Supporting Information). Notably, SgII_1–161_, without phase separation, had a reduction effect similar to SgII‐KD, while SgII_1–479_, with complete phase separation, had a rescue effect similar to full‐length SgII_1–617_ (WT) (Figure 5F,H and Figure S6D,E, Supporting Information), fully suggesting that SgII phase separation is pivotal in regulating the size of LDCV and consequently influencing QS in ACCs.

Next, we investigated how SgII phase separation contributes to the size of LDCV. As we know, matrix proteins are packaged in the trans‐Golgi network (TGN) before LDCV forming,^[^ ^68^ ^]^ and the TGN contains high concentration of calcium ion (3–5 mM Ca^2+^).^[^ ^70^ ^]^ Therefore, combining our previous data that SgII can undergo phase separation in cells (Figure 4I–J), we speculate that SgII phase droplets can be formed in TGN and then bud at TGN membrane, ultimately contributing to LDCV size. To verify our hypothesis, following the mixture of SgII‐mcherry and Ca^2+^ to form the phase droplets, we added the 25% (v/v) reconstituted bio‐lipids (tagged with green fluorescent molecule NBD and dissolved in ethanol buffer)^[^ ^75^, ^76^ ^]^ into the droplets‐containing solution, and clearly observed the generation of green circular bio‐lipids structures surrounding around those SgII droplets (Figure 5I and Figure S7A–C, Supporting Information). To rule out that the presence of ethanol may drastically affect the experimental outcome, we found that ethanol did not alter SgII phase droplets, and lipids alone (without SgII and Ca^2+^) were totally dissolved in ethanol and did not form green circular structures (Figure S7D,E, Supporting Information). In addition, as a negative control, the kind of surrounding circular structures was observed neither in SgII‐mCherry nor Ca^2+^ alone with lipids (Figure S7F,G, Supporting Information).

Next, we purified SgII‐mCherry at pH 6.5 (a physiological pH of TGN^[^ ^77^ ^]^) and found that SgII still formed phase droplets with Ca^2+^ at pH 6.5 (Figure S7H,I, Supporting Information), suggesting SgII phase separation in TGN is possible. Another question is whether the SgII concentration in native ACCs is suitable to undergo phase separation like that in vitro. To measure the native SgII concentration, we collected SgII protein from mice adrenal medullas and fitted the Western blot results to the quantitative curve between the weight of SgII purified in vitro and its fluorescence intensity (Figure S7J). The results showed that the native SgII concentration in ACCs was 1.05 ± 4.0 mg g^−1^ (about 20 µM) (Figure S7K, Supporting Information), which was ≈4 times higher than the minimal concentration required for SgII phase separation in vitro (5 µM, Figure S4E, Supporting Information). Taken together, we believe SgII can undergo phase separation in native conditions and directly influences LDCV size at the first formation step, providing enough volume for LDCV synthesis by budding at TGN membrane, and sequentially enlarges the size of newly synthesized LDCV (Figure 5J).

### 2.6 Endogenous SgIII Regulates LDCV Size and QS by Influencing SgII Phase Separation in ACCs

**Figure 6:**
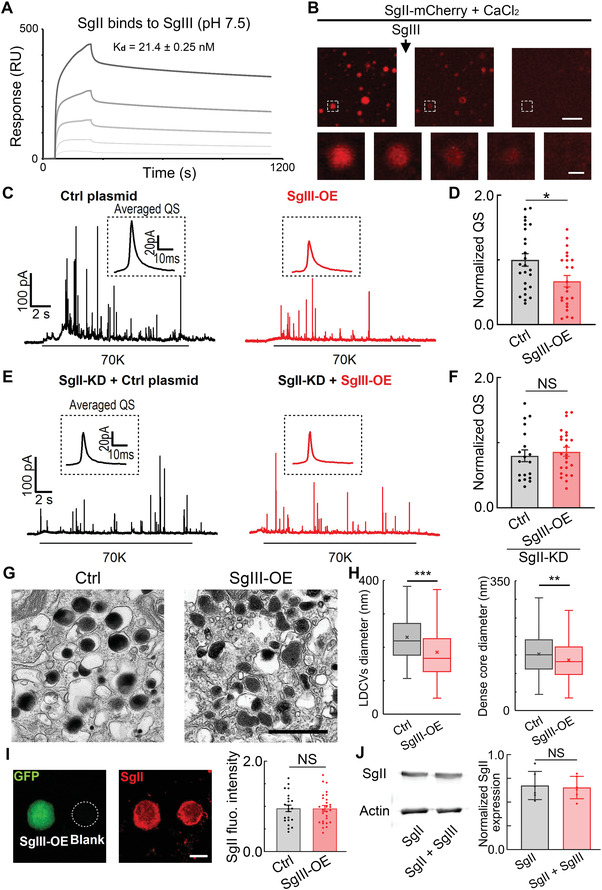
SgIII regulates LDCV size and QS by affecting SgII phase separation in ACCs. A) SPR analysis of the binding affinity between SgII and SgIII at pH 7.5. The calculated dissociation constant (Kd) value at pH 7.5 is 21.4 nM, indicating that SgII binds to SgIII. B) Upper panel, SgIII (SgII‐mCherry:SgIII (molar ratio) = 1:4) abolishes SgII phase droplets (scale bar, 40 µm). Lower panel, the selected SgII droplet (dotted box) gradually disappears after adding SgIII (scale bar, 2 µm). C) Representative amperometric recordings of catecholamine release from control and SgIII‐OE ACCs. D) Corresponding to panel (C), statistics of normalized QS in control and SgIII‐OE ACCs (*n* = 25 for control ACCs and 23 for SgIII‐OE ACCs, unpaired Student's *t*‐test). E) Representative amperometric recordings of catecholamine release from control and SgIII‐OE in SgII‐KD ACCs. F) Corresponding to panel (E), statistics of normalized QS in control and SgIII‐OE cells after SgII‐KD (*n* = 19 for control ACCs and 24 for SgIII‐OE ACCs, unpaired Student's *t*‐test). G) Representative transmission EM micrographs of LDCVs in control and SgIII‐OE ACCs (scale bar, 500 nm). H) Histograms showing that SgIII‐OE leads to a reduction of LDCV diameter ≈19% and dense core diameter ≈12%. Statistics are from 241 LDCVs in control and 306 in SgIII‐OE ACCs (K–S test). I) Left, immunostaining of SgII expression in control and SgIII‐OE ACCs (scale bar, 10 µm). Right, statistics of SgII fluorescent intensity from the left panel (*n* = 22 cells per group, unpaired Student's *t*‐test), indicating that SgIII‐OE does not alter the SgII expression level in native ACCs. J) Left, representative western blots of SgII in transfected Cos‐7 cells. Right, statistics of normalized SgII expression from the left panel (*n* = 6 independent experiments, paired Student's *t*‐test), indicating that SgIII‐OE does not alter the SgII expression level in cell lines. Data are presented as the mean ± SEM with scatter dots (D,F,I,J) or the medians with interquartile ranges (H). *P < 0.05, **P < 0.01, ***P < 0.001; NS, no significant difference.

To verify the importance of SgII phase separation in the regulation of LDCV size, we searched for a physiological modulator that may participate in SgII phase separation and then regulate related functions. Secretogranin III (SgIII), another secretogranin family member, is known to be weakly expressed in the adrenal gland^[^ ^78^ ^]^ and serves as a SgII binding partner.^[^ ^79^ ^]^ The DNA of SgIII was successfully cloned from mouse adrenal medulla cDNA (Figure S8A, Supporting Information) and the protein was purified in high purity (Figure S8B, Supporting Information). Then, we tested the binding ability between SgIII and SgII. The SPR results showed that the *K*_d_ value for SgIII and SgII was quite strong, 21.4 nM at pH 7.5 (**Figure** **6**A), consistent with the previous study.^[^ ^79^ ^]^ Next, we investigated whether the strong interaction between SgIII and SgII affects the phase separation of SgII. Surprisingly, SgII‐mCherry droplets dissipated when SgIII was added (Figure 6B, Figure S8C and Movie S7, Supporting Information). Remarkably, the degree of dissipation of SgII droplets depended on the amount of SgIII added: more SgIII resulted in faster and more thorough dissipation (Figure 6B and Figure S8 C,E, Supporting Information). As a control to avoid the dilution effect, mCherry alone did not destroy the SgII droplets (Figure S8D,E, Supporting Information). Based on the molecular determinants in protein phase separation,^[^ ^80^ ^]^ we infer that SgII phase droplets are formed by hydrophobic intermolecular interactions, and SgIII abolishes these interactions by competitively binding to SgII.

Since SgIII abolished SgII phase separation in vitro, we continued to investigate whether the phase separation‐mediated functions of SgII in native ACCs are also affected by SgIII. To mimic the dissipative effect of SgIII on SgII phase separation, a plasmid overexpressing SgIII (SgIII‐OE) was designed and transfected into ACCs by electroporation. Immunofluorescence data confirmed that SgIII was successfully overexpressed in native ACCs and endogenous SgIII was weakly detectable in control cells (Figures S7J and S9A,B, Supporting Information). CFE recordings showed that the QS of catecholamine released from the LDCVs of SgIII‐OE ACCs was significantly reduced by ≈35% compared with control cells (Figure 6C,D and Figure S9C,E, Supporting Information). Meanwhile, SgIII‐OE did not affect the quantal release in SgII‐KD ACCs (Figure 6E,F and Figure S9D,F, Supporting Information), indicating that the quantal release reduction by SgIII‐OE was dependent on endogenous SgII. Next, CLEM was used to select the transfected ACCs and image LDCVs in control and SgIII‐OE cells (Figure 6G). The diameters of LDCVs and dense cores were reduced by ≈20% and ≈10%, respectively, in SgIII‐OE ACCs compared with control cells (Figure 6H and Figure S9G, Supporting Information). Thus, SgIII‐OE, SgII_1–161_‐rescue, and SgII‐KD had similar effects on both the QS and physical size of LDCVs in ACCs (Figures 1C–G, 2H–J, 5E–H, and 6C–H.), proving that SgIII counters the size‐determining function of SgII, the phase separation, in LDCVs. Furthermore, to determine whether SgIII‐OE influences the size of LDCVs and QS by disrupting phase separation but not by decreasing the SgII expression level in ACCs, we applied immunofluorescent staining for SgII. The results showed that the expression of SgII was almost the same in control and SgIII‐OE ACCs (Figure 6I and Figure S9H, Supporting Information). Meanwhile, to confirm this conclusion, we overexpressed SgII and SgIII in Cos‐7 cells. Western blot analysis showed that SgIII‐OE did not alter the expression level of SgII in this cell line as well (Figure 6J). These results together suggest that SgIII reduces the size of LDCVs by binding to SgII, sequentially changing the SgII phase separation level but not the SgII expression level, finally resulting in QS reduction. In summary, combining thorough studies of the endogenous SgII phase separation modulator SgIII (Figure 6) and previous SgII truncations with different phase separation abilities (Figure 5), we conclude that SgII phase separation is the determining factor in ACCs, contributing to LDCV size and quantal neurotransmitter release.

## 3 Discussion

**Figure 7:**
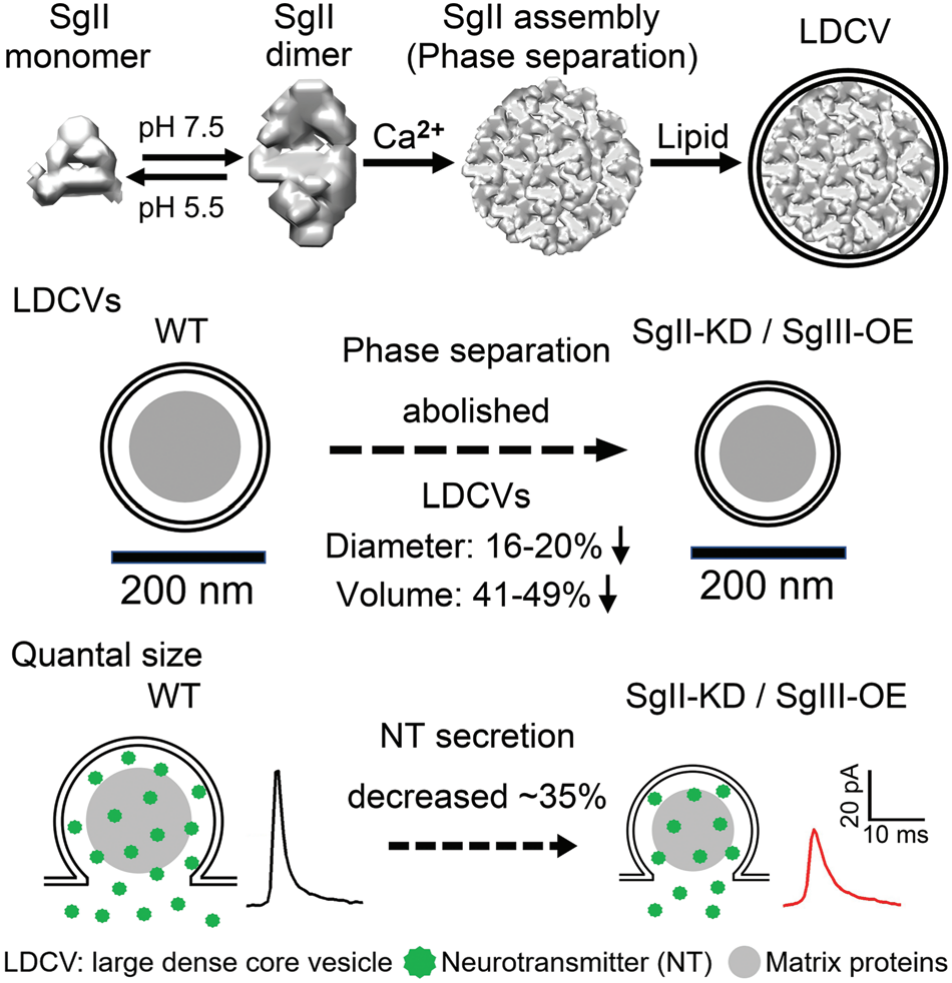
The mechanism of SgII liquid–liquid phase separation determining LDCV size and quantal release in mammalian neuroendocrine cells.

The vesicle size determines the appropriate amount of neurotransmitter loaded into the vesicle for synaptic transmission and related physiological functions.^[^ ^49^, ^50^, ^81^ ^]^ However, the underlying molecular mechanisms for determining the size of LDCVs are still poorly understood. In the present work, we have uncovered that SgII, a vesicle matrix protein, is critical to determine LDCV size and quantal neurotransmitter release by phase separation in ACCs (**Figure** **7**).

The first finding of this work was that LDCV quantal release of neurotransmitters (including catecholamine and neuropeptide) was strikingly decreased by using shRNAs targeting SgII, and this reduction could be fully rescued by SgII overexpression in ACCs (Figure 1 and Figure S1, Supporting Information). Compared with the other major intravesicular matrix proteins CgA and CgB (accounting for ≈90% of the matrix protein in LDCVs), SgII accounted for only 1% of the matrix protein,^[^ ^24^ ^]^ but SgII‐KD resulted in a 35% reduction in QS, which was comparable with CgA‐KO (≈40% QS reduction) or CgB‐KO (≈33% QS reduction).^[^ ^26^, ^34^ ^]^ This suggests that SgII is an important element in regulating LDCV release in ACCs. It is known that QS is regulated by the vesicle fusion pore and intravesicular catecholamine‐binding matrix protein CgA in ACCs.^[^ ^26^ ^]^ However, our data showed that 1) SgII‐KD had no effects on the fusion pore size (Figures 2A–C and 1I) and SOM‐induced sub‐quantal release via fusion pore shrinkage (Figure 2D–F), strongly proving that SgII is not a fusion pore regulator, and 2) SgII bound to catecholamine with extremely low affinity (Figure S2E,F, Supporting Information), which was quite different from CgA, a protein that strongly binds to catecholamine and contributes to the sub‐quantal release of LDCVs in ACCs.^[^ ^26^, ^29^ ^]^ Therefore, the mechanism by which SgII regulates quantal release in LDCVs may be a novel mode, quite different from that of CgA.

So what is the mechanism by which SgII regulates the QS of LDCVs? By using CLEM, we were surprised to find that the diameter of LDCVs was significantly reduced ≈16% by SgII‐KD in ACCs (Figure 2H–J); converted to volume, this is a ≈40% reduction, consistent with the change in QS (Figure 1E and Figure S2H, Supporting Information). Although the QS of ACCs is reduced in CgA‐KO, CgB‐KO, and CgA/B double gene‐knockout mice,^[^ ^12^, ^26^, ^28^, ^33^, ^35^ ^]^ the current conclusion that CgA and CgB affect the LDCV size in ACCs is still ambiguous. It has been reported that LDCV size is decreased in some CgA‐KO mice,^[^ ^31^, ^35^ ^]^ while it is increased in some other CgA antisense transgenic mice and CgA/B double gene‐knockout mice^[^ ^30^, ^33^ ^]^ and remains unchanged in some other reports of CgA‐KO mice.^[^ ^12^, ^32^ ^]^ Therefore, CgA and CgB are probably not the key proteins to determine LDCV size. In our work, we demonstrated that SgII is the key regulator of LDCV size in native ACCs, which is also consistent with the previous report that depletion of SgII expression leads to a decrease in vesicle size in PC12 cells.^[^ ^82^ ^]^

By introducing biochemical and structural biology methods, we revealed how a small amount of the matrix protein SgII can determine the size of vesicles. We found that 1) SgII formed polymers at Ph 7.5 (Figure 3A–E); 2) the polymerized SgII had a filamentous structure, arranged in a dimer‐dimer conjunct double‐stranded helix (Figure 3F,G and Figure S3C,D,Supporting Information); 3) the polymerized SgII formed droplets via liquid–liquid phase separation in both buffer solutions and cells (Figure 4 and Figure S4, Supporting Information); 4) SgII phase separation depends on the length of protein and SgII truncations with impaired phase separation resulted in smaller LDCVs and less quantal catecholamine release (Figure 5C–H and Figures S5 and S6, Supporting Information); 5) SgII phase droplets can recruit bio‐lipid (Figure 5I and Figure S7, Supporting Information); 6) SgIII abolished SgII phase separation, and SgIII‐OE in native ACCs reduced vesicle QS ≈35% and reduced LDCV size ≈19% (Figure 6, Figures S8 and S9A–G, Supporting Information), similar to the SgII‐KD effect in ACCs (Figures 1 and 2H–J); and 7) the effect of SgIII‐OE on quantal vesicle release was mediated by SgII (Figure 6C–F, and Figure S9C–F, Supporting Information) via altering SgII phase separation but not the SgII expression level (Figure 6I,J and Figure S9H, Supporting Information). These data together suggest that SgII serves as an LDCV scaffold protein in ACCs to determine vesicle size and affect QS (Figure 7). Our future plan may be to uncover the structure of SgII at much finer and higher resolution, to explore its key residues in phase separation. Besides, the unique structural characteristics of filamentous SgII at pH 7.5 (Figure 3F,G) support the hypothesis that it is a vesicle scaffold protein, using its helical structure to expand the volume of LDCV, just like the architecture of the cell scaffold proteins actin and tubulin.^[^ ^83^ ^]^

SgII formed liquid–liquid phase separation with proper Ca^2+^ or other divalent cations at pH 7.5, but not at pH 5.5 (Figure 4). As SgII is an acidic protein and has a high content of negatively charged glutamic acid and aspartic acid residues,^[^ ^18^ ^]^ Ca^2+^ probably reduces the Debye length, allowing stronger interactions to overcome the electrostatic repulsion and promoting phase separation.^[^ ^84^, ^85^ ^]^ In a PEG‐simulated crowded environment that mimics the intracellular environment, SgII forms droplets with 1 mM Ca^2+^, which is close to the physiological Ca^2+^ concentration in the secretory pathway‐related organelles, ER, and Golgi complex.^[^ ^69^, ^70^ ^]^ Combining the findings that: 1) SgII formed phase separation in vivo in cell lines (Figure 4I–J); 2) its truncations with different phase separation abilities tuned the LDCV size and quantal release in ACCs (Figure 5C–H and Figures S5 and S6, Supporting Information); 3) its binding ability with reconstituted bio‐lipid (Figure 5I and Figure S7A–G, Supporting Information) and 4) the reasonable pH value and concentration for SgII phase separation formation in TGN (Figure S7H–K, Supporting Information), we believe that SgII forms phase separation in TGN or even in ER, consequently enlarging LDCV size at an early stage and further providing more spaces for catecholamines and neuropeptides storage in mature LDCVs, resulting in more neurotransmitter release. In the future, it may be possible to image SgII phase separation in ACCs and identify the precise location where SgII phase separation happens under physiological conditions.

Furthermore, SgIII, an endogenous SgII‐binding protein (Figure 6A),^[^ ^79^ ^]^ reduced quantal vesicle release and LDCV size via regulating the function (phase separation) of SgII in native ACCs (Figure 6 and Figures S8 and S9, Supporting Information). We speculate that SgII and SgIII may serve as counterparts or chaperones to regulate the size of LDCVs in ACCs (or other tissues containing LDCVs, such as the midbrain monoamine nuclei, hypothalamus, pituitary gland, and pancreatic islets) to tune the secretion level of LDCV cargos (neurotransmitters or neuromodulators) to adapt to changing physiological or pathological conditions.^[^ ^86^ ^]^

To conclude, SgII is conserved in different species (Figure S10, Supporting Information), and LDCVs are critical for neuropeptide and hormone release in many endocrine and neuroendocrine cells as well as neurons to regulate many important physiological processes.^[^ ^87^, ^88^, ^89^, ^90^ ^]^ In the present work, we provide sufficient experiments to prove the causal relationship between protein liquid–liquid phase separation and the regulation of LDCV size and neurotransmitters quantal release. Protein phase separation is considered to be a new feature for some proteins and it plays a vital role in many physiological processes.^[^ ^54^, ^59^ ^]^ By combining experimental methods from different fields (physiology/electrochemistry and biochemistry/structural biology), considerable evidence here revealed a new function of SgII as a scaffold protein to determine LDCV size via liquid–liquid phase separation, subsequently regulating quantal neurotransmitter release, which may be a universal phenomenon in LDCVs in different cells from various species.

## 4 Experimental Section

### Animals and Chemicals

C57BL/6 mice were from Beijing Vital River Laboratory Animal Technology Co., Ltd. All procedures and animal care were approved by the Institutional Animal Care and Use Committee of Peking University (Beijing, China) and the Association for Assessment and Accreditation of Laboratory Animal Care (zhouz11). Adult mice (C57BL/6 strain, 2–4 months old, both sexes) were used for all experiments. Mice were housed under a 12‐h light/dark cycle with food and water. All chemicals were from Sigma unless otherwise stated.

### Plasmid Preparation

The nucleotide target sequences GGA GCA GTT CTG CTT CTT ATC (SgII‐shRNA1) and GGA CAC GCC TGA TGA CTA TGA (SgII‐shRNA2) were chosen to silence the expression of mouse SgII. A random sequence (TTC TCC GAA CGT GTC ACG T) that was predicted to target no genes in mouse cells served as a negative control (scrambled) (Guangzhou RiboBio Co.). Annealed double‐stranded oligonucleotides encoding the target sequences were inserted into the vector p‐RNAT‐H1.1‐RFP/GFP to generate plasmids expressing shRNAs against SgII. An RNAi (SgII‐shRNA1)‐resistant form of mouse SgII for the rescue experiments was generated by introducing the following silent mutations: GGC GCG GTA CTA CTC. The coding sequences of mouse SgII (NM_009129.3), SgII truncations (SgII_1–479_, SgII_1–325, 480–617_, SgII_1–161, 326–617_, SgII_1–30, 162–617_, SgII_1–325_, SgII_1–30, 326–617_, SgII_1–161_, SgII_1–30, 162–325_, SgII_1–30, 326–479_, SgII_1–30, 480–617_, and SgII_31–617_), and SgIII (NM_001164790.1) were amplified by single‐step PCR from mouse adrenal cDNA and subcloned into a pEG BacMam expression vector with mCherry/EGFP and 10× His‐tag attached to the C terminus for protein purification experiments. SgII, SgII truncations (SgII_1–479_, SgII_1–325_, and SgII_1–161_), and SgIII were also cloned into the pIRES2‐EGFP (Clontech) plasmid for SgII‐rescue and SgIII‐overexpression experiments in ACCs.

### ACC Preparation and Transfection

Mice ACCs were prepared as described previously.^[^ ^26^ ^]^ Briefly, the adrenal glands were isolated from anesthetized animals (25% ethyl carbamate), then the cortex was removed, cut into pieces, and digested by papain enzyme for 40 min at 37 °C under 5% CO_2_. The pieces were then triturated gently through a 200‐µL pipette tip. After centrifugation, the cells were plated on coverslips pre‐coated with 0.1% poly‐L‐lysine, incubated at 37 °C under 5% CO_2_, and used within 24–96 h. Chromaffin cells were transfected for genetic manipulations using a 10‐µL Neon electroporation system (Invitrogen, MPK1096) according to the manufacturer instruction.

### Immunofluorescence

Chromaffin cells and Cos‐7 cells were prefixed in 4% paraformaldehyde (PFA) for 20 min, washed three times with phosphate‐buffered saline (PBS), then permeabilized with 0.3% Triton in bovine serum albumin (BSA) for 3 min. After the cells were incubated with 2% BSA for 1 h, they were incubated with the primary antibodies against SgII (GeneTex, GTX116446) and SgIII (Proteintech, 10954‐1‐AP) overnight at 4 °C. Then, the cells were washed with PBS and incubated with the secondary antibody (Alexa Fluor 594 goat anti‐rabbit IgG, A11037, Invitrogen) for 1 h. After that, the cells were washed with PBS and stained with DAPI. Finally, cells were mounted on coverslips immersed in 50% glycerol. Fluorescence micrographs were acquired on a confocal microscope (Zeiss LSM 710) and analyzed with ImageJ (NIH) software.

### Western Blot

Transfected Cos‐7 cells or ACCs were lysed in RIPA (C1053, Applygen) with proteinase inhibitor cocktail for 30 min on ice and centrifuged at 15 000 g for 30 min. Equal amounts of supernatant were then separated on 10% SDS‐PAGE and blotted onto polyvinylidene difluoride membranes (Merck Millipore). The primary antibodies were for SgII (GeneTex, GTX116446) and actin (A5316, Sigma); the secondary antibodies were IRDye 800CW goat anti‐rabbit IgG (LIC‐926‐3221, LI‐COR Biosciences) and IRDye 680CW goat anti‐mouse IgG (LIC‐926‐32220, LI‐COR Biosciences). The protein bands were then imaged under an infrared scanner (Odyssey, Gene Co.).

### Electrochemistry

Highly sensitive, low‐noise, 7 µm CFEs (ProCFE, Dagan) were used for the electrochemical monitoring of quantal catecholamine release from single chromaffin cells. An EPC10/2 amplifier with Patchmaster software was used to record the CFE signal.^[^ ^41^, ^91^ ^]^ The normal extracellular buffer was composed of (in mM): 145 NaCl, 2 CaCl_2_, 2.8 KCl, 1 MgCl_2_, and 10 HEPES (pH 7.4). The high‐potassium (70 K) solution contained (in mM) 78 NaCl, 2 CaCl_2_, 70 KCl, 1 MgCl_2_, and 10 HEPES (pH 7.4). All experiments were performed at room temperature unless otherwise indicated.

### TIRF Image and Analysis

TIRF imaging was performed on an inverted microscope with a 100 TIRF objective lens (Olympus IX‐81; numerical aperture 1.45). Images were captured by an Andor EMCCD using Andor iQ software with an exposure time of 53 ms (see also described previously.^[^ ^45^, ^92^ ^]^) The standard bath solution contained the following (in mM): 145 NaCl, 2.8 KCl, 2 CaCl2, 1 MgCl2·6H_2_O, 10 H‐HEPES, and 10 D‐glucose, pH 7.4. The high‐K stimulus contained the following (in mM): 85 NaCl, 70 KCl, 2.5 CaCl2, 1 MgCl2·6H_2_O, 10 H‐HEPES, and 10 D‐glucose, pH 7.4, and was applied using a gravity perfusion system (RCP‐2B, Yibo). Mouse ACCs were co‐transfected with NPY‐pHluorin and scrambled/SgII‐KD plasmids and were cultured for 4 days before TIRF imaging experiments. Exocytotic events were defined as abrupt fluorescence increases, immediately followed by a fluorescence decrease or diffusion of NPY‐pHluorin puncta in the vicinity. Fluorescent intensity was calculated and analyzed using ImageJ; the intensity values during the 0.5‐s baseline before the peak value were averaged and used as F0.

### TMR‐Dextran Uptake

Cultured mouse ACCs were washed 3 times with cold standard extracellular bath solution, then incubated for 2 min with 50 µM dextran (10 and 40 kDa, Invitrogen, D1816, and D1842, respectively) in 37 °C 70 mM KCl containing external solution. Unbound dye was washed out with standard cold 0 Ca^2+^ immediately after incubation. The cells were then incubated in cold 0 Ca^2+^ solution. Z‐series of 1‐µm optical sections were scanned through the 40×oil‐immersion lens of a Zeiss 710 inverted confocal microscope. The consecutive optical sections were z‐projected and the total numbers of dextran fluorescent puncta per cell were determined. Images were processed with ImageJ (NIH).

### Baculovirus Recombination and Protein Purification

Bacmids were generated by transfecting plasmids into DH10Bac *Escherichia coli* cells, and recombinant baculoviruses were produced by Sf9 insect cells with Cellfectin II reagents (Life Technologies). 6 days after transfection, low‐titer baculovirus stock was obtained and used for further amplification. Proteins used for cryo‐EM, SPR analysis, AUC, and phase separation assays were expressed in the BacMam system.^[^ ^93^, ^94^, ^95^ ^]^ High‐titer baculovirus was used to infect HEK293S cells at a density of 2 × 10^−6^ cells mL^−1^ in Yocon HEK293 medium supplemented with 10% FBS (at 37 °C and under 5% CO_2_). After 8–12 h, 10 mM sodium butyrate was added, and the cell culture was maintained in suspension for 66–72 h. Cells were then collected by centrifugation at 6000 × g for 20 min, re‐suspended in lysis buffer (25 mM Tris pH 7.5, 500 mM NaCl) containing protease inhibitors (1 µg mL^−1^ aprotinin, 1 µg mL^−1^ leupeptin, 1 µg mL^−1^ pepstatin, 20 µg mL^−1^ trypsin inhibitor, 1 mM benzamidine, and 1 mM phenylmethylsulfonyl fluoride) and DNase (2 µg mL^−1^). The re‐suspended cells were disrupted by ultrasonication for 20 min at 4 °C. After lysates were removed by centrifugation at 18 000 × g for 20 min, the supernatant was collected and incubated with Ni^2+^‐NTA agarose (DiNing) for 2 h at 4 °C. His‐mCherry‐tagged proteins were eluted with a gradient of imidazole‐containing high‐salt Tris‐buffered saline (TBS, 25 mM Tris pH 7.5, 500 mM NaCl). Non‐tagged proteins were obtained by incubation with PreScission protease at 4 °C overnight to separate the His‐mCherry from the protein. After that, proteins were run on a Superose 6 Increase 10/300 GL column (GE Healthcare) in different buffers (25 mM Tris pH 7.5, 500 mM NaCl; 25 mM Na‐Citrate pH 6.5, 500 mM NaCl; 25 mM NaAc/HAc pH 5.5, 500 mM NaCl) as needed for further purification and applied to SDS‐PAGE for analysis.

### CLEM and EM

Transfected ACCs were cultured on 35‐mm glass‐bottomed dishes with a positive marker (MatTek Corp., P35G‐1.5‐14‐C‐GRID). After 3 days of culture for SgII‐KD and 1 day for SgIII‐OE, successfully transfected cells were spotted by EGFP/mCherry under a fluorescence microscope. After selection, cells were fixed with 2% glutaraldehyde plus 2% PFA for 1 h at room temperature and post‐fixed by osmium tetroxide with 1% aqueous uranyl acetate. The cells were then dehydrated through a graded alcohol series and embedded in SPI‐Pon 812 resin (SPI Supplies, PA). Subsequently, sections were cut at 70 nm on an ultramicrotome (Leica Microsystem, UC7) and a 120‐kV transmission electron microscope (JEOL, JEM‐1400) was used in all EM imaging.

### Cryo‐EM Sample Preparation, Data Collection, and Processing

Purified SgII was concentrated at 4.5 mg mg^−1^ and incubated at 4 °C for 2 days (providing enough time for filament formation). Then, 3‐µL samples were placed onto glow‐discharged Quantifoil R1.2/1.3 400 Au holey carbon grids (Quantifoil), blotted in Vitrobot (FEI) using a 2‐s blotting time, and then plunge‐frozen in liquid ethane. Cryo‐EM images were captured on a 300‐kV Titan Krios (FEI) microscope with a Gatan imaging filter (20 eV) and a K2 summit detector (Gatan). The physical pixel size was 1.055 Å and the dose rate was 8 electrons per pixel per s. 1840 images of SgII filaments were collected. Image motion was corrected by MotionCor2^[^ ^96^ ^]^ and contrast transfer function was estimated by Gctf.^[^ ^97^ ^]^ Other processing steps were performed in RELION 3.1.^[^ ^98^ ^]^ All particles were manually picked and boxed out into 20 Å with 50% overlap between neighbors from one filament. 2D classification was used to select good classes and 3D refinement was used to generate an initial model based on a featureless cylinder. Several rounds of 3D refinement of the large dataset led to a final stable resolution with the symmetry parameters of Δ*Φ* = 12.0° and Δ*z* = 100.3 Å.

### Analytical Ultracentrifugation (AUC)

Sedimentation velocity assays were performed on a Beckman Optima AUC at 4 °C. Protein samples were prepared to 280‐nm absorption at 1.0–400 µL, loaded into a conventional double‐sector quartz cell, and mounted in a Beckman four‐hole An‐60 Ti rotor. Data were collected every 60 s at a wavelength of 280 nm at 45000 rpm for 8 h. Sedimentation velocity data were used to calculate the interference sedimentation coefficient distributions by SEDFIT software.

### SPR Analysis

All SPR assays were performed on a BiacoreT200 (GE Healthcare) instrument at 25 °C as described previously.^[^ ^94^ ^]^ Purified SgII was immobilized on the surface of a CM5 sensor chip using the amine‐coupling method. Two different pH running buffers (25 mM Tris pH 7.5, 150 mM NaCl; 25 mM NaAc/HAc pH 5.5, 150 mM NaCl) were supplemented with 0.05% P20. To measure the catecholamine binding affinity of SgII, NE was injected as analyte in serial twofold dilutions at concentrations ranging from 0.2 µM to 500 mM. The flow‐rate was 30 µL min^−1^ for 60 s association and 60 s dissociation, and the dissociation constant was calculated in kinetic mode. To measure the binding affinity of SgII with SgIII, SgIII was injected as analyte in serial twofold dilutions at concentrations ranging from 2 to 1000 nM. The flow‐rate was 30 µL min^−1^ for 180 s association and 900 s dissociation. In this case, a flow‐rate of 30 µL min^−1^ for 30 s of 10 mM glycine (pH 2.5) regeneration was required between different analyte injections to bring the signal back to baseline. The dissociation constant was calculated in affinity mode.

### Phase Separation Assay

SgII phase separation assays were performed with the indicated buffers and ions at 25 °C on a Zeiss LSM 710 confocal microscope. Proteins with or without other components were first mixed in a test tube, and then pipetted onto 35‐mm coverslips. Wavelengths of 488 and 561 nm were used for EGFP and mCherry excitation, respectively. For FRAP experiments, 561‐nm laser light was used at maximum intensity, at 2 Hz for 5 s. For experiments with addition of 1,6‐hexanediol and SgIII, SgII‐mCherry was first incubated with Ca^2+^ for 3 min and then 1,6‐hexanediol and SgIII were added to the solution. Fields of view were randomly selected from each individual experiment. All images were analyzed by ImageJ (NIH) software. For experiments with addition of lipid, lipid was mixed by DSPC (250 µM), DOPE (150 µM), SoyPI (50 µM), DMB PEG (5 µM), and NBD PC (5 µM) in ethanol. After 3 min incubation of SgII‐mCherry and Ca^2+^ for 3 min, lipid was added at the volume ratio of 1:3 (25% ethanol).

### Statistical Analysis

All experiments were replicated at least three times. Data were presented as the mean ± SEM (standard error of the mean) or the medians with interquartile ranges. “*n*” presents the number of independent events as reported in the figure legends. Paired or unpaired Student's *t*‐test, one‐way ANOVA, or the Kolmogorov–Smirnov (K–S) test were chosen for data statistics after normality testing. All tests were conducted using Prism V7.0 (GraphPad Software, Inc.). Statistical tests were two‐tailed and the level of significance was set at *P* < 0.05 (*P < 0.05; **P < 0.01; ***P < 0.001; NS, no significant difference).

## Conflict of Interest

The authors declare no conflict of interest.

## Author Contributions

Z.L., Y.L., and Y.H. contributed equally to this work. Q.F.Z. and Z.Z. designed the research; Z.H.L., Y.L.L., and Y.Q.H performed experiments and analyzed data; C.H.W., B.L., J.L., L.L.Y., X.H.J., X.Y.D., Z.J.Q., and F.P.Z. performed experiments; Q.F.Z., Z.Z., and Z.Z. wrote the paper.

## Supporting information

Supporting Information

Supplemental Movie 1

Supplemental Movie 2

Supplemental Movie 3

Supplemental Movie 4

Supplemental Movie 5

Supplemental Movie 6

Supplemental Movie 7

## Data Availability

The data that support the findings of this study are available from the corresponding author upon reasonable request.

## References

[1] P. De Camilli , R. Jahn , Annu. Rev. Physiol. 1990, 52, 625. 2184771 10.1146/annurev.ph.52.030190.003205

[2] H. Park , M. M. Poo , Nat. Rev. Neurosci. 2013, 14, 7. 23254191 10.1038/nrn3379

[3] A. N. van den Pol , Neuron 2012, 76, 98. 23040809 10.1016/j.neuron.2012.09.014 PMC3918222

[4] C. M. Persoon , A. Moro , J. P. Nassal , M. Farina , J. H. Broeke , S. Arora , N. Dominguez , J. R. van Weering , R. F. Toonen , M. Verhage , EMBO J. 2018, 37, 20. 10.15252/embj.201899672 PMC6187028 30185408

[5] A. Meyer‐Lindenberg , G. Domes , P. Kirsch , M. Heinrichs , Nat. Rev. Neurosci. 2011, 12, 524. 21852800 10.1038/nrn3044

[6] J. F. McGinty , T. W. Whitfield Jr. , W. J. Berglind , Brain Res. 2010, 1314, 183. 19732758 10.1016/j.brainres.2009.08.078 PMC2819624

[7] L. H. Vahatalo , S. T. Ruohonen , L. Ailanen , E. Savontaus , Neuropeptides 2016, 55, 31. 26681068 10.1016/j.npep.2015.11.088

[8] Z. P. Pang , T. C. Sudhof , Curr. Opin. Cell Biol. 2010, 22, 496. 20561775 10.1016/j.ceb.2010.05.001 PMC2963628

[9] A. G. Garcia , A. M. Garcia‐De‐Diego , L. Gandia , R. Borges , J. Garcia‐Sancho , Physiol. Rev. 2006, 86, 1093. 17015485 10.1152/physrev.00039.2005

[10] R. H. Chow , L. von Ruden , E. Neher , Nature 1992, 356, 60. 1538782 10.1038/356060a0

[11] A. Albillos , G. Dernick , H. Horstmann , W. Almers , G. Alvarez de Toledo , M. Lindau , Nature 1997, 389, 509. 9333242 10.1038/39081

[12] M. S. Montesinos , J. D. Machado , M. Camacho , J. Diaz , Y. G. Morales , D. Alvarez de la Rosa , E. Carmona , A. Castaneyra , O. H. Viveros , D. T. O'Connor , S. K. Mahata , R. Borges , J. Neurosci. 2008, 28, 3350. 18367602 10.1523/JNEUROSCI.5292-07.2008 PMC6670582

[13] H. Winkler , E. Westhead , Neuroscience 1980, 5, 1803. 7432623 10.1016/0306-4522(80)90031-7

[14] J. Santodomingo , L. Vay , M. Camacho , E. Hernandez‐Sanmiguel , R. I. Fonteriz , C. D. Lobaton , M. Montero , A. Moreno , J. Alvarez , Eur J Neurosci 2008, 28, 1265. 18973554 10.1111/j.1460-9568.2008.06440.x

[15] H. Kasai , N. Takahashi , H. Tokumaru , Physiol. Rev. 2012, 92, 1915. 23073634 10.1152/physrev.00007.2012

[16] J. Estevez‐Herrera , A. Gonzalez‐Santana , R. Baz‐Davila , J. D. Machado , R. Borges , J. Neurochem. 2016, 137, 897. 26990968 10.1111/jnc.13609

[17] E. Crivellato , B. Nico , D. Ribatti , Anat. Rec. 2008, 291, 1587. 10.1002/ar.20763 19037853

[18] L. Taupenot , K. L. Harper , D. T. O'Connor , N. Engl. J. Med. 2003, 348, 1134. 12646671 10.1056/NEJMra021405

[19] K. B. Helle , M. H. Metz‐Boutigue , M. C. Cerra , T. Angelone , Pflugers Arch 2018, 470, 143. 28875377 10.1007/s00424-017-2027-6

[20] S. A. Feldman , L. E. Eiden , Endocr. Pathol. 2003, 14, 3. 12746559 10.1385/ep:14:1:3

[21] P. Banks , K. Helle , Biochem. J. 1965, 97, 40C. 10.1042/bj0970040c PMC1264782 5881651

[22] R. W. Lee , W. B. Huttner , J. Biol. Chem. 1983, 258, 11326. 6577005

[23] P. Rosa , A. Zanini , Mol. Cell. Endocrinol. 1981, 24, 181. 7297761 10.1016/0303-7207(81)90058-7

[24] S. H. Yoo , FASEB J. 2010, 24, 653. 19837865 10.1096/fj.09-132456 PMC2830129

[25] J. S. Videen , M. S. Mezger , Y. M. Chang , D. T. O'Connor , J. Biol. Chem. 1992, 267, 3066. 1737762

[26] Q. Zhang , B. Liu , Q. Wu , B. Liu , Y. Li , S. Sun , Y. Wang , X. Wu , Z. Chai , X. Jiang , X. Liu , M. Hu , Y. Wang , Y. Yang , L. Wang , X. Kang , Y. Xiong , Y. Zhou , X. Chen , L. Zheng , B. Zhang , C. Wang , F. Zhu , Z. Zhou , Neuron 2019, 102, 173. 30773347 10.1016/j.neuron.2019.01.031

[27] G. Wen , S. K. Mahata , P. Cadman , M. Mahata , S. Ghosh , N. R. Mahapatra , F. Rao , M. Stridsberg , D. W. Smith , P. Mahboubi , N. J. Schork , D. T. O'Connor , B. A. Hamilton , Am. J. Hum. Genet. 2004, 74, 197. 14740315 10.1086/381399 PMC1181918

[28] J. Diaz‐Vera , Y. G. Morales , J. R. Hernandez‐Fernaud , M. Camacho , M. S. Montesinos , F. Calegari , W. B. Huttner , R. Borges , J. D. Machado , J. Neurosci. 2010, 30, 950. 20089903 10.1523/JNEUROSCI.2894-09.2010 PMC6633114

[29] R. Westermann , F. Stogbauer , K. Unsicker , R. Lietzke , FEBS Lett. 1988, 239, 203. 3181426 10.1016/0014-5793(88)80917-7

[30] T. Kim , C. F. Zhang , Z. Sun , H. Wu , Y. P. Loh , Neuroscience 2005, 25, 6958. 16049171 10.1523/JNEUROSCI.1058-05.2005 PMC6724839

[31] N. R. Mahapatra , D. T. O'Connor , S. M. Vaingankar , A. P. Hikim , M. Mahata , S. Ray , E. Staite , H. Wu , Y. Gu , N. Dalton , B. P. Kennedy , M. G. Ziegler , J. Ross , S. K. Mahata , J. Clin. Invest. 2005, 115, 1942. 16007257 10.1172/JCI24354 PMC1159140

[32] G. N. Hendy , T. Li , M. Girard , R. C. Feldstein , S. Mulay , R. Desjardins , R. Day , A. C. Karaplis , M. L. Tremblay , L. Canaff , Mol. Endocrinol. 2006, 20, 1935. 16556729 10.1210/me.2005-0398

[33] J. Diaz‐Vera , M. Camacho , J. D. Machado , N. Domínguez , M. S. Montesinos , J. R. Hernández‐Fernaud , R. Luján , R. Borges , FASEB J. 2011, 26, 430. 21990378 10.1096/fj.11-181941

[34] J. Estevez‐Herrera , M. R. Pardo , N. Dominguez , D. Pereda , J. D. Machado , R. Borges , Biomol. Concepts 2013, 4, 605. 25436760 10.1515/bmc-2013-0020

[35] T. Pasqua , S. Mahata , G. K. Bandyopadhyay , A. Biswas , G. A. Perkins , A. P. Sinha‐Hikim , D. S. Goldstein , L. E. Eiden , S. K. Mahata , Cell Tissue Res. 2016, 363, 693. 26572539 10.1007/s00441-015-2316-3 PMC5529139

[36] M. G. Cozzi , A. Zanini , Cell Biol. Int. Rep. 1988, 12, 493. 3401941 10.1016/0309-1651(88)90141-5

[37] S. H. Yoo , S. Y. Chu , K. D. Kim , Y. H. Huh , Biochemistry 2007, 46, 14663. 18020452 10.1021/bi701339m

[38] J. Del Castillo , B. Katz , J. Physiol. 1954, 124, 560. 13175199 10.1113/jphysiol.1954.sp005129 PMC1366292

[39] J. E. Lisman , S. Raghavachari , R. W. Tsien , Nat. Rev. Neurosci. 2007, 8, 597. 17637801 10.1038/nrn2191

[40] Z. Zhou , S. Misler , R. H. Chow , Biophys. J. 1996, 70, 1543. 8785312 10.1016/S0006-3495(96)79718-7 PMC1225082

[41] R. M. Wightman , J. A. Jankowski , R. T. Kennedy , K. T. Kawagoe , T. J. Schroeder , D. J. Leszczyszyn , J. A. Near , E. J. Diliberto Jr. , O. H. Viveros , Proc. Natl. Acad. Sci. U. S. A. 1991, 88, 10754. 1961743 10.1073/pnas.88.23.10754 PMC53009

[42] E. Ales , L. Tabares , J. M. Poyato , V. Valero , M. Lindau , G. Alvarez de Toledo , Nat. Cell Biol. 1999, 1, 40. 10559862 10.1038/9012

[43] T. Blackmer , E. C. Larsen , C. Bartleson , J. A. Kowalchyk , E. J. Yoon , A. M. Preininger , S. Alford , H. E. Hamm , T. F. Martin , Nat. Neurosci. 2005, 8, 421. 15778713 10.1038/nn1423

[44] X. K. Chen , L. C. Wang , Y. Zhou , Q. Cai , M. Prakriya , K. L. Duan , Z. H. Sheng , C. Lingle , Z. Zhou , Nat. Neurosci. 2005, 8, 1160. 16116443 10.1038/nn1529

[45] Q. Wu , Q. Zhang , B. Liu , Y. Li , X. Wu , S. Kuo , L. Zheng , C. Wang , F. Zhu , Z. Zhou , J. Neurosci. 2019, 39, 199. 30381405 10.1523/JNEUROSCI.1255-18.2018 PMC6360287

[46] A. Elhamdani , H. C. Palfrey , C. R. Artalejo , Neuron 2001, 31, 819. 11567619 10.1016/s0896-6273(01)00418-4

[47] W. Shin , L. Ge , G. Arpino , S. A. Villarreal , E. Hamid , H. Liu , W. D. Zhao , P. J. Wen , H. C. Chiang , L. G. Wu , Cell 2018, 173, 934. 29606354 10.1016/j.cell.2018.02.062 PMC5935532

[48] H. Fathali , J. Dunevall , S. Majdi , A. S. Cans , ACS Chem. Neurosci. 2017, 8, 368. 27966899 10.1021/acschemneuro.6b00350

[49] P. S. Pinheiro , A. M. Jansen , H. de Wit , B. Tawfik , K. L. Madsen , M. Verhage , U. Gether , J. B. Sorensen , J. Neurosci. 2014, 34, 10688. 25100601 10.1523/JNEUROSCI.5132-13.2014 PMC4122802

[50] L. W. Gong , I. Hafez , G. Alvarez de Toledo , M. Lindau , J. Neurosci. 2003, 23, 7917. 12944522 10.1523/JNEUROSCI.23-21-07917.2003 PMC6740609

[51] S. F. Banani , H. O. Lee , A. A. Hyman , M. K. Rosen , Nat. Rev. Mol. Cell Biol. 2017, 18, 285. 28225081 10.1038/nrm.2017.7 PMC7434221

[52] L. P. Bergeron‐Sandoval , N. Safaee , S. W. Michnick , Cell 2016, 165, 1067. 27203111 10.1016/j.cell.2016.05.026

[53] Y. Shin , C. P. Brangwynne , Science 2017, 357, 6357. 10.1126/science.aaf4382 28935776

[54] P. Li , S. Banjade , H. C. Cheng , S. Kim , B. Chen , L. Guo , M. Llaguno , J. V. Hollingsworth , D. S. King , S. F. Banani , P. S. Russo , Q. X. Jiang , B. T. Nixon , M. K. Rosen , Nature 2012, 483, 336. 22398450 10.1038/nature10879 PMC3343696

[55] V. H. Ryan , N. L. Fawzi , Trends Neurosci. 2019, 42, 693. 31493925 10.1016/j.tins.2019.08.005 PMC6779520

[56] F. G. Quiroz , V. F. Fiore , J. Levorse , L. Polak , E. Wong , H. A. Pasolli , E. Fuchs , Science 2020, 367, eaax9554. 32165560 10.1126/science.aax9554 PMC7258523

[57] G. Zhu , J. Xie , W. Kong , J. Xie , Y. Li , L. Du , Q. Zheng , L. Sun , M. Guan , H. Li , T. Zhu , H. He , Z. Liu , X. Xia , C. Kan , Y. Tao , H. C. Shen , D. Li , S. Wang , Y. Yu , Z. H. Yu , Z. Y. Zhang , C. Liu , J. Zhu , Cell 2020, 183, 490. 33002410 10.1016/j.cell.2020.09.002 PMC7572904

[58] G. Zhang , Z. Wang , Z. Du , H. Zhang , Cell 2018, 174, 1492. 30173914 10.1016/j.cell.2018.08.006

[59] D. Milovanovic , Y. Wu , X. Bian , P. De Camilli , Science 2018, 361, 604. 29976799 10.1126/science.aat5671 PMC6191856

[60] A. Boija , I. A. Klein , B. R. Sabari , A. Dall'Agnese , E. L. Coffey , A. V. Zamudio , C. H. Li , K. Shrinivas , J. C. Manteiga , N. M. Hannett , B. J. Abraham , L. K. Afeyan , Y. E. Guo , J. K. Rimel , C. B. Fant , J. Schuijers , T. I. Lee , D. J. Taatjes , R. A. Young , Cell 2018, 175, 1842. 30449618 10.1016/j.cell.2018.10.042 PMC6295254

[61] M. Zeng , Y. Shang , Y. Araki , T. Guo , R. L. Huganir , M. Zhang , Cell 2016, 166, 1163. 27565345 10.1016/j.cell.2016.07.008 PMC5564291

[62] A. C. Murthy , W. S. Tang , N. Jovic , A. M. Janke , D. H. Seo , T. M. Perdikari , J. Mittal , N. L. Fawzi , Nat. Struct. Mol. Biol. 2021, 28, 923. 34759379 10.1038/s41594-021-00677-4 PMC8654040

[63] N. Beuret , H. Stettler , A. Renold , J. Rutishauser , M. Spiess , J. Biol. Chem. 2004, 279, 20242. 14996840 10.1074/jbc.M310613200

[64] M. D. Whim , J. Neurosci. 2006, 26, 6637. 16775152 10.1523/JNEUROSCI.5100-05.2006 PMC6674048

[65] D. Perrais , I. C. Kleppe , J. W. Taraska , W. Almers , J Physiol 2004, 560, 413. 15297569 10.1113/jphysiol.2004.064410 PMC1665250

[66] C. M. Persoon , R. I. Hoogstraaten , J. P. Nassal , J. R. T. van Weering , P. S. Kaeser , R. F. Toonen , M. Verhage , Neuron 2019, 104, 1065. 31679900 10.1016/j.neuron.2019.09.015 PMC6923582

[67] K. L. Lynch , R. R. Gerona , D. M. Kielar , S. Martens , H. T. McMahon , T. F. Martin , Mol. Biol. Cell 2008, 19, 5093. 18799625 10.1091/mbc.E08-03-0235 PMC2592635

[68] T. Kim , M. C. Gondre‐Lewis , I. Arnaoutova , Y. P. Loh , Physiology 2006, 21, 124. 16565478 10.1152/physiol.00043.2005

[69] J. F. Sambrook , Cell 1990, 61, 197. 2184940 10.1016/0092-8674(90)90798-j

[70] M. Bendayan , J. Roth , A. Perrelet , L. Orci , J. Histochem. Cytochem. 1980, 28, 149. 7354212 10.1177/28.2.7354212

[71] B. R. Sabari , A. Dall'Agnese , A. Boija , I. A. Klein , E. L. Coffey , K. Shrinivas , B. J. Abraham , N. M. Hannett , A. V. Zamudio , J. C. Manteiga , C. H. Li , Y. E. Guo , D. S. Day , J. Schuijers , E. Vasile , S. Malik , D. Hnisz , T. I. Lee , I. I. Cisse , R. G. Roeder , P. A. Sharp , A. K. Chakraborty , R. A. Young , Science 2018, 361, 6400. 10.1126/science.aar3958 PMC6092193 29930091

[72] S. S. Patel , B. J. Belmont , J. M. Sante , M. F. Rexach , Cell 2007, 129, 83. 17418788 10.1016/j.cell.2007.01.044

[73] K. Ribbeck , D. Gorlich , EMBO J. 2002, 21, 2664. 12032079 10.1093/emboj/21.11.2664 PMC126029

[74] T. M. Perdikari , A. C. Murthy , N. L. Fawzi , bioRxiv 2022.

[75] S. Sugiura , J. Mima , Sci. Rep. 2016, 6, 20407. 26838333 10.1038/srep20407 PMC4738300

[76] L. V. Boldyreva , M. V. Morozova , S. S. Saydakova , E. N. Kozhevnikova , Int. J. Mol. Sci. 2021, 22, 21. 10.3390/ijms222111682 PMC8584226 34769112

[77] A. Deschamps , A. S. Colinet , O. Zimmermannova , H. Sychrova , P. Morsomme , Sci. Rep. 2020, 10, 1881. 32024908 10.1038/s41598-020-58795-w PMC7002768

[78] L. Han , M. Suda , K. Tsuzuki , R. Wang , Y. Ohe , H. Hirai , T. Watanabe , T. Takeuchi , M. Hosaka , Mol. Endocrinol. 2008, 22, 1935. 18483175 10.1210/me.2008-0006 PMC2505326

[79] K. Hotta , M. Hosaka , A. Tanabe , T. Takeuchi , J. Endocrinol. 2009, 202, 111. 19357184 10.1677/JOE-08-0531

[80] S. Boeynaems , S. Alberti , N. L. Fawzi , T. Mittag , M. Polymenidou , F. Rousseau , J. Schymkowitz , J. Shorter , B. Wolozin , L. Van Den Bosch , P. Tompa , M. Fuxreiter , Trends Cell Biol. 2018, 28, 420. 29602697 10.1016/j.tcb.2018.02.004 PMC6034118

[81] L. He , L. Xue , J. Xu , B. D. McNeil , L. Bai , E. Melicoff , R. Adachi , L. G. Wu , Nature 2009, 459, 93. 19279571 10.1038/nature07860 PMC2768540

[82] M. Courel , A. Soler‐Jover , J. L. Rodriguez‐Flores , S. K. Mahata , S. Elias , M. Montero‐Hadjadje , Y. Anouar , R. J. Giuly , D. T. O'Connor , L. Taupenot , J. Biol. Chem. 2010, 285, 10030. 20061385 10.1074/jbc.M109.064196 PMC2843166

[83] B. Bugyi , M. Kellermayer , J. Muscle Res. Cell Motil. 2020, 41, 3. 31093826 10.1007/s10974-019-09515-z PMC7109165

[84] A. Agarwal , S. K. Rai , A. Avni , S. Mukhopadhyay , Proc. Natl. Acad. Sci. U. S. A. 2021, 118, 45. 10.1073/pnas.2100968118 PMC8609423 34737230

[85] A. M. Smith , A. A. Lee , S. Perkin , J. Phys. Chem. Lett. 2016, 7, 2157. 27216986 10.1021/acs.jpclett.6b00867

[86] X. Zhan , G. Wen , E. Jiang , F. Li , X. Wu , H. Pang , J. Toxicol. Sci. 2020, 45, 271. 32404559 10.2131/jts.45.271

[87] K. Mitchell , M. Mikwar , D. Da Fonte , C. Lu , B. Tao , D. Peng , W. Erandani , W. Hu , V. L. Trudeau , Gen. Comp. Endocrinol. 2020, 299, 113588. 32828813 10.1016/j.ygcen.2020.113588

[88] I. Splawski , K. W. Timothy , L. M. Sharpe , N. Decher , P. Kumar , R. Bloise , C. Napolitano , P. J. Schwartz , R. M. Joseph , K. Condouris , H. Tager‐Flusberg , S. G. Priori , M. C. Sanguinetti , M. T. Keating , Cell 2004, 119, 19. 15454078 10.1016/j.cell.2004.09.011

[89] L. He , X. S. Wu , R. Mohan , L. G. Wu , Nature 2006, 444, 102. 17065984 10.1038/nature05250

[90] F. Torrealba , M. A. Carrasco , Brain Res. Rev. 2004, 47, 5. 15572159 10.1016/j.brainresrev.2004.06.004

[91] Z. Zhou , S. Misler , Proc. Natl. Acad. Sci. U. S. A. 1995, 92, 6938. 7624348 10.1073/pnas.92.15.6938 PMC41446

[92] Y. Wang , Q. Wu , M. Hu , B. Liu , Z. Chai , R. Huang , Y. Wang , H. Xu , L. Zhou , L. Zheng , C. Wang , Z. Zhou , Sci. Signaling 2017, 10. 10.1126/scisignal.aal1683 28634208

[93] A. Dukkipati , H. H. Park , D. Waghray , S. Fischer , K. C. Garcia , Protein Expression Purif. 2008, 62, 160. 10.1016/j.pep.2008.08.004 PMC2637115 18782620

[94] Z. Lin , J. Liu , H. Ding , F. Xu , H. Liu , Nat. Commun. 2018, 9, 268. 29348579 10.1038/s41467-017-02414-2 PMC5773555

[95] Y. Jiang , T. Liu , C. H. Lee , Q. Chang , J. Yang , Z. Zhang , Nature 2020, 588, 658. 33053563 10.1038/s41586-020-2862-z

[96] S. Q. Zheng , E. Palovcak , J. P. Armache , K. A. Verba , Y. Cheng , D. A. Agard , Nat. Methods 2017, 14, 331. 28250466 10.1038/nmeth.4193 PMC5494038

[97] K. Zhang , J. Struct. Biol. 2016, 193, 1. 26592709 10.1016/j.jsb.2015.11.003 PMC4711343

[98] R. Fernandez‐Leiro , S. H. W. Scheres , Acta Crystallogr., Sect. D: Struct. Biol. 2017, 73, 496. 28580911 10.1107/S2059798316019276 PMC5458491

