# Supplementary material for: Tuning the Size of Large Dense‐Core Vesicles and Quantal Neurotransmitter Release via Secretogranin II Liquid–Liquid Phase Separation
Source: Adv Sci (Weinh). 2022 Jul 27;9(27):2202263. doi: 10.1002/advs.202202263 (PMC9507364; doi:10.1002/advs.202202263)
Supplement: Supplementary file 1 — Supporting Information [file ADVS-9-2202263-s005.pdf]

ADVANCED SCIENCE
Open Access

Supporting Information

for *Adv. Sci.*, DOI 10.1002/advs.202202263

Tuning the Size of Large Dense-Core Vesicles and Quantal Neurotransmitter Release via Secretogranin II Liquid–Liquid Phase Separation

Zhaohan Lin, Yinglin Li, Yuqi Hang, Changhe Wang, Bing Liu, Jie Li, Lili Yin, Xiaohan Jiang, Xingyu Du, Zhongjun Qiao, Feipeng Zhu, Zhe Zhang, Quanfeng Zhang* and Zhuan Zhou**

Supplemental Information

Tuning the size of large dense-core vesicles and quantal neurotransmitter release *via* secretogranin II liquid-liquid phase separation

Zhaohan Lin[#], Yinglin Li[#], Yuqi Hang[#], Changhe Wang, Bing Liu, Jie Li, Lili Yin, Xiaohan Jiang, Xingyu Du, Zhongjun Qiao, Feipeng Zhu, Zhe Zhang*, Quanfeng Zhang*, Zhuan Zhou*

State Key Laboratory of Membrane Biology and Beijing Key Laboratory of Cardiometabolic Molecular Medicine, Institute of Molecular Medicine, College of Future Technology, Peking University and Peking-Tsinghua Center for Life Sciences and PKU-IDG/McGovern Institute for Brain Research, Peking University, Beijing 100871, China.

#Equal contributors

*Corresponding author (Zhuan Zhou: zzhou@pku.edu.cn; Quanfeng Zhang: zhangqf@pku.edu.cn; Zhe Zhang: zzhang01@pku.edu.cn)

Supplementary Figures: S1-S10

Supplemental Movies: S1-S7

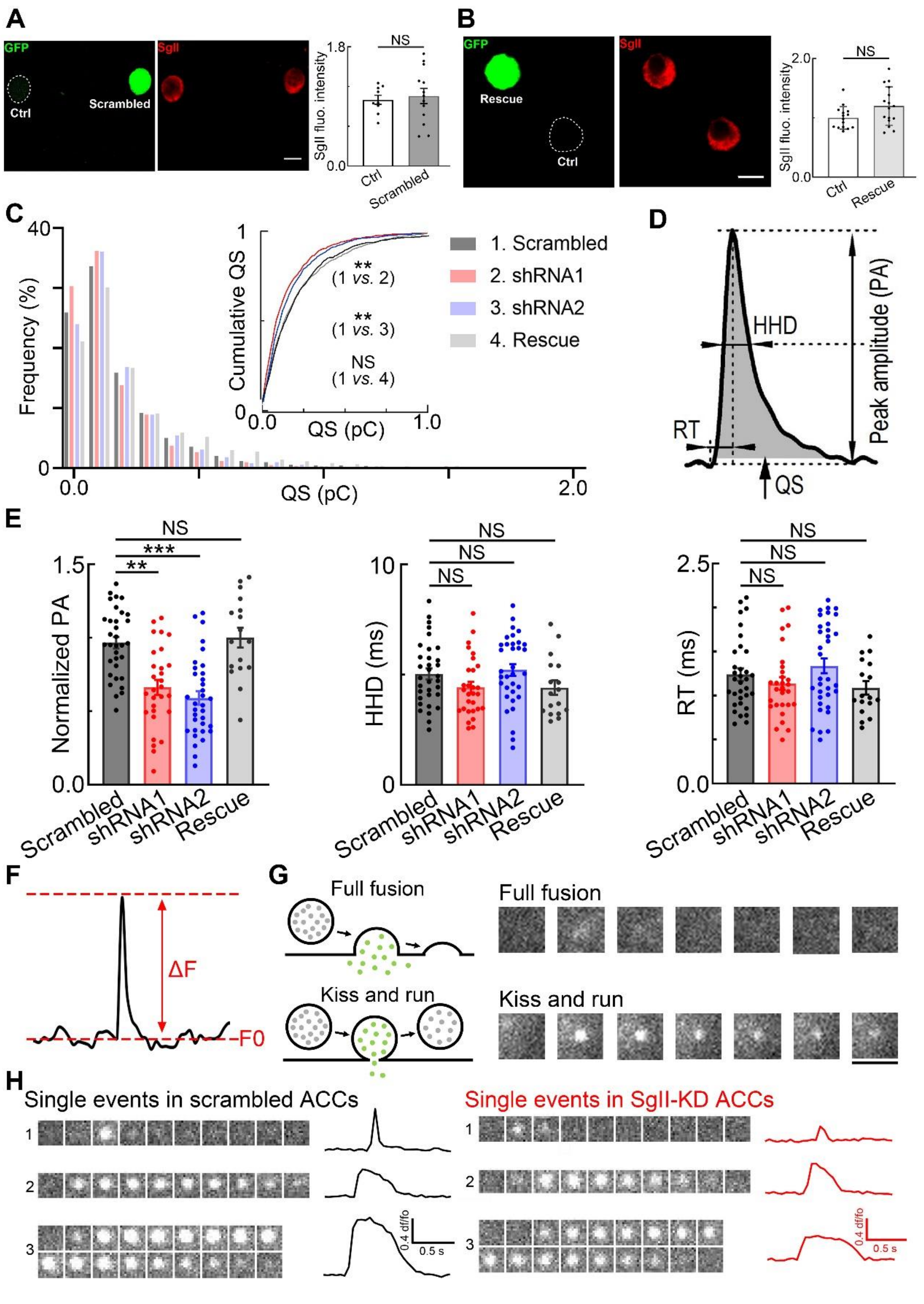

Figure S1 (related to Figure 1). Quantal neurotransmitter release is reduced by SgII-KD in ACCs by CFE and TIRF methods.

(A and B) SgII expression level in ACCs is successfully rescued by shRNA1-resistant-SgII. For (A), typical SgII immunostaining images (left) and the

corresponding statistics (right) in ctrl and scrambled ACCs (scale bar, 10 μm) (n = 9 ctrl and 13 scrambled cells, unpaired Student's *t* test). The scrambled plasmids do not alter the expression of SgII. For (B), similar as (A), but in ctrl and shRNA1-resistant-SgII rescue ACCs (scale bar, 10 μm) (n = 14 ctrl and 14 rescued cells, unpaired Student's *t* test). The shRNA1-resistant-SgII rescue plasmids rescued the expression of SgII to the control level.

(C) Frequency distribution and cumulative curve of QS in scrambled, SgII-KD, and rescued ACCs corresponding to Figure 1E (Kolmogorov-Smirnov test).

(D) Definition of the kinetics variables peak amplitude (PA), half-height duration (HHD), rise-time (RT), and quantal size (QS) of a single fusion event by CFE.

(E) Quantitative analyses of PA, HHD, and RT of amperometric spikes from scrambled, SgII-KD, and rescued cells by CFE (n = 33 for scrambled cells, 29 cells for shRNA1, 34 cells for shRNA2, and 17 for rescued cells; one-way ANOVA).

(F) The definition of F0 (baseline) and ΔF (increase of fluorescence) in TIRF image.

(G) Two vesicular fusion modes in ACCs by TIRF imaging. Upper, a "full fusion" event: with a spreading fluorescence, corresponding to full release of neurotransmitters via a larger fusion pore. Lower, a "kiss and run" event: only a brief fluorescence brightening in the release center, corresponding to partial release of neurotransmitters via a smaller fusion pore (see also [1]).

(H) Three typical single LDCV fusion events from NPY-pHluorin transfected scrambled (left) and SgII-KD (right) ACCs by real time TIRF imaging. Image size, 1 μm × 1 μm; 53 ms per frame. Data are presented as the mean ± SEM with scatter dots (A, B, and E). $^{**}P < 0.01$, $^{***}P < 0.001$; NS, no significant difference.

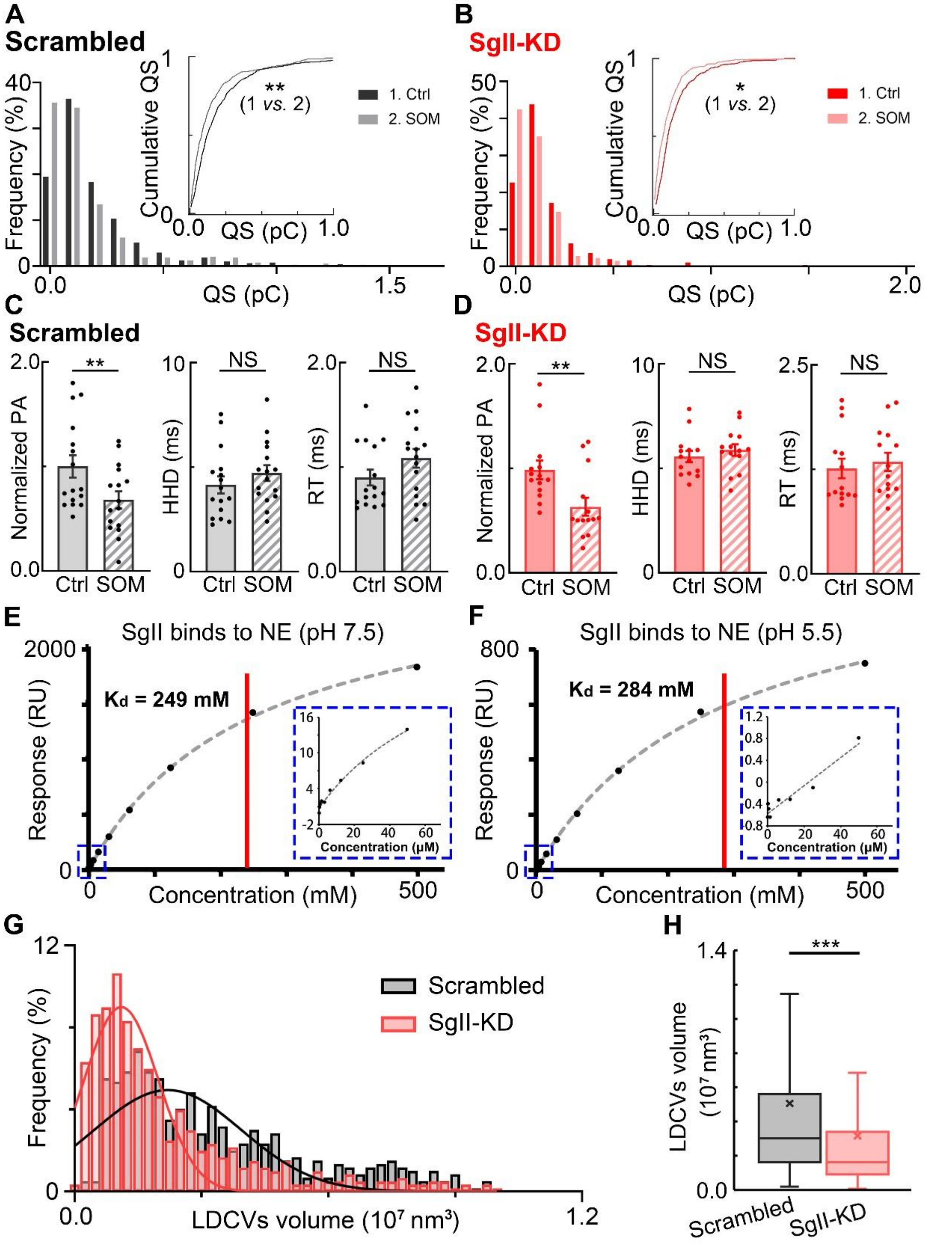

Figure S2 (related to Figure 2). The statistics of SOM effect on quantal release and the SPR analysis of binding affinity between SgII and NE.

(A) Frequency distribution and cumulative curve of QS in scrambled ACCs before and after SOM treatment, corresponding to Figure 2E (Kolmogorov-Smirnov test).

(B) Same as (A), but in SgII-KD ACCs, corresponding to Figure 2F.

(C) Peak amplitude (PA) is reduced by somatostatin (SOM, 500 nM) in scrambled

ACCs (n = 16, paired Student's *t* test), but the half-height duration (HHD) and rise time (RT) remain unchanged.

(D) Same as (C), but in SgII-KD ACCs (n = 14, paired Student's *t* test).

(E and F) Surface plasma resonance (SPR) analysis of the binding affinity between SgII and catecholamine (NE) at pH 7.5 (E) and 5.5 (F) (dotted box, enlargement of low concentrations of NE). The calculated dissociation constant (*Kd*) values between SgII and NE at pH 7.5 and pH 5.5 are 249 mM and 284 mM, respectively.

(G) Distribution of LDCVs volume in scrambled and SgII-KD ACCs, corresponding to Figure 2I.

(H) The statistic of LDCVs volume in scrambled and SgII-KD ACCs, corresponding to panel (G) (Kolmogorov-Smirnov test). Data are presented as the mean ± SEM with scatter dots (C and D) or the medians with interquartile ranges (H). *P < 0.05, **P < 0.01, ***P < 0.001, NS, no significant difference.

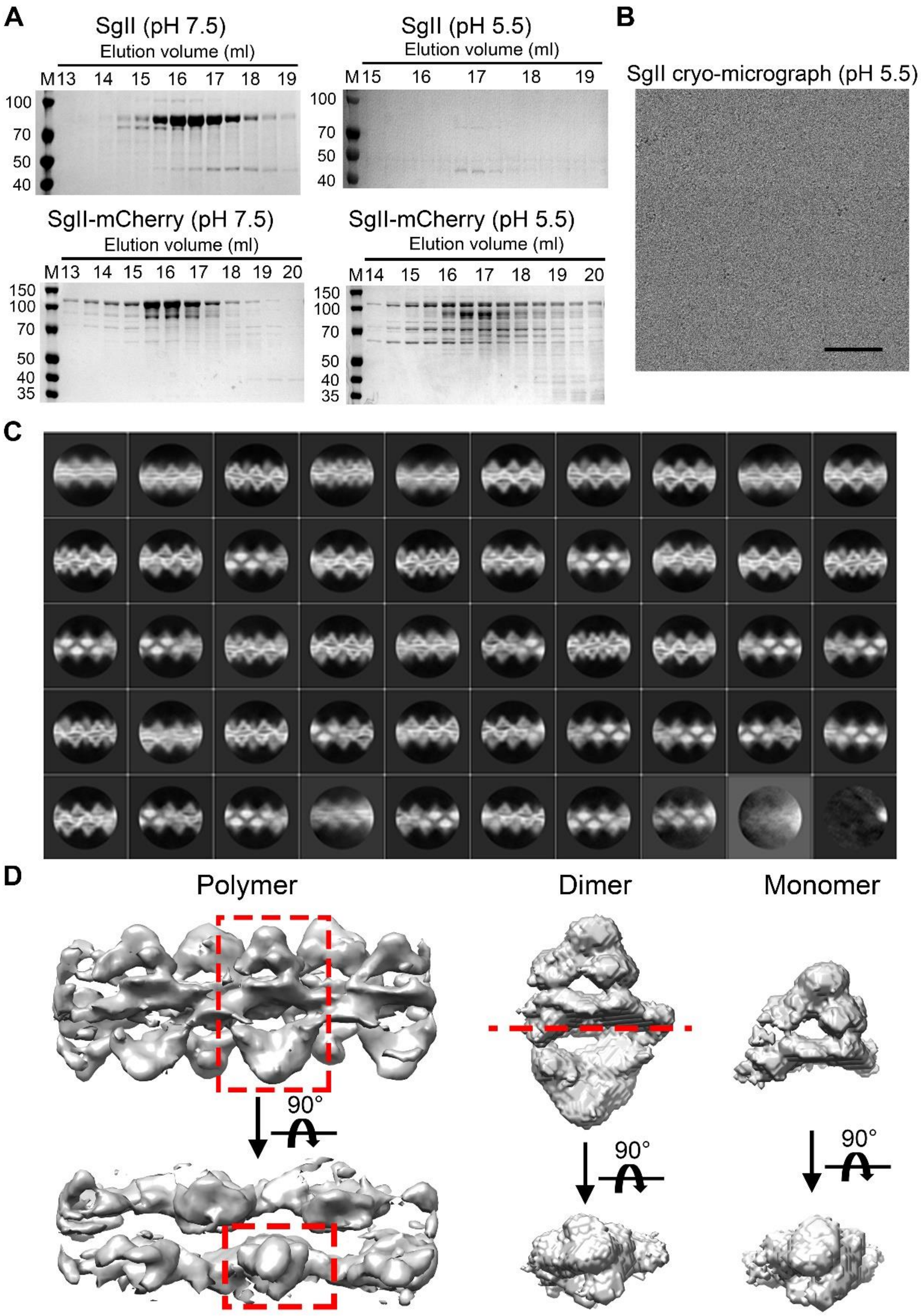

Figure S3 (related to Figure 3). SDS-PAGE and Cryo-EM data processing results of SgII.

(A) Corresponding SDS-PAGE of SgII and SgII-mCherry purification in size exclusion chromatography at pH 7.5 and pH 5.5.

(B) Representative cryo-electron micrograph of SgII at pH 5.5. Scale bar, 100 nm.

(C) SgII filament fragments are classified by two-dimensional (2D) classification into 50 classes, sorted by distribution.

(D) Dimeric and monomeric SgII subunits are extracted from filament structure and showed in the magnified view. One dimeric subunit is enclosed by red dashed lines in the polymer panel. The axis of symmetry is shown by the red dashed line in the dimer panel.

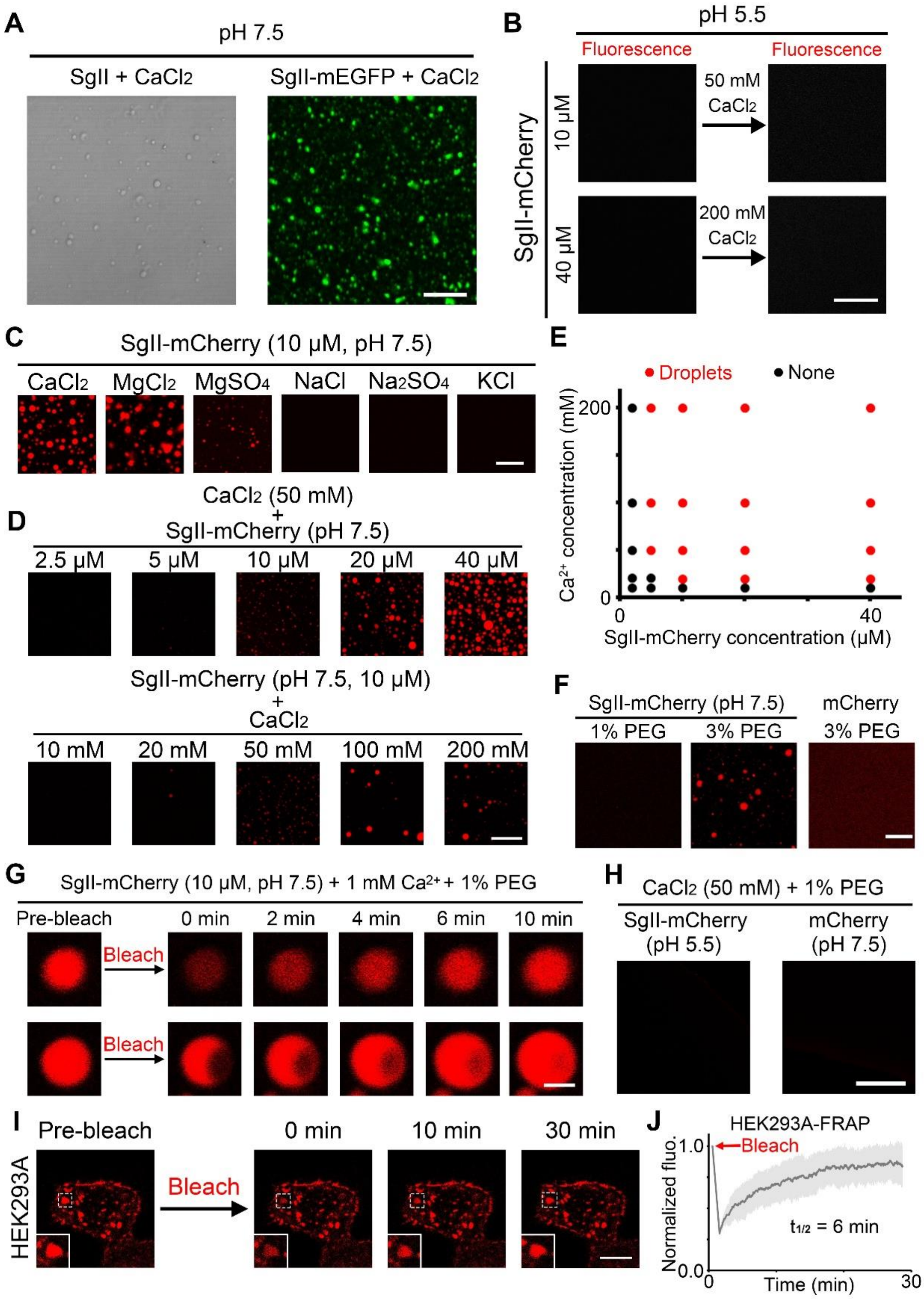

Figure S4 (related to Figure 4). SgII phase separation is dependent on pH, divalent cations, SgII and PEG concentrations *in vitro* and SgII also undergoes phase seperation in HEK293A cells *in vivo*.

(A) SgII without fluorescent tag and SgII tagged with monomeric EGFP (mEGFP) still form phase droplets with Ca^{2+} in the bright field and fluorescent field,

respectively, indicating that they are formed by SgII itself and not the tagged proteins (scale bar, 20 μm).

(B) SgII (10 μM or 40 μM) do not form droplets at pH 5.5 even with 50 mM or 200 mM Ca^{2+} (scale bar, 40 μm).

(C) SgII droplets are induced by the divalent cations Ca^{2+} and Mg^{2+} at pH 7.5, but not the univalent cations Na^{+} and K^{+}, or the anions Cl^{-} and SO_4^{2-} (scale bar, 20 μm).

(D) Upper panel, SgII phase separation showing dependence on its own concentration. Fluorescence micrographs of SgII-mCherry with gradient concentrations from 2.5 to 40 μM in a 50 mM Ca^{2+} buffer. Lower panel, SgII phase separation showing dependence on Ca^{2+} concentration. Fluorescence micrographs of 10 μM SgII-mCherry with indicated gradient Ca^{2+} concentrations from 10 to 200 mM (scale bar, 40 μm).

(E) The relationship between the phase separation of SgII and Ca^{2+} (red dots, SgII forms phase separation; black dots, SgII does not undergo phase separation; scale bar, 40 μm).

(F) SgII-mCherry can form droplets in 3% PEG but not 1% PEG (w/v). The mCherry cannot form droplets even in 3% PEG (w/v) (scale bar, 20 μm).

(G) Fully (upper) and partially (lower) photo-bleached SgII-mCherry droplets can recover their fluorescence in 1% PEG environment with 1 mM Ca^{2+} (scale bar, 3 μm).

(H) SgII-mCherry at pH 5.5 and mCherry alone at pH 7.5 do not form droplets with 50 mM Ca^{2+} plus 1% PEG (w/v) (scale bar, 40 μm).

(I) FRAP in HEK293A cells: fluorescence intensity of fully photobleached $SgII_{chim}$ granular structure recovers within 30 min (scale bar, 10 μm).

(J) Statistics of FRAP experiments corresponding to panel (I) (n = 7 cells).

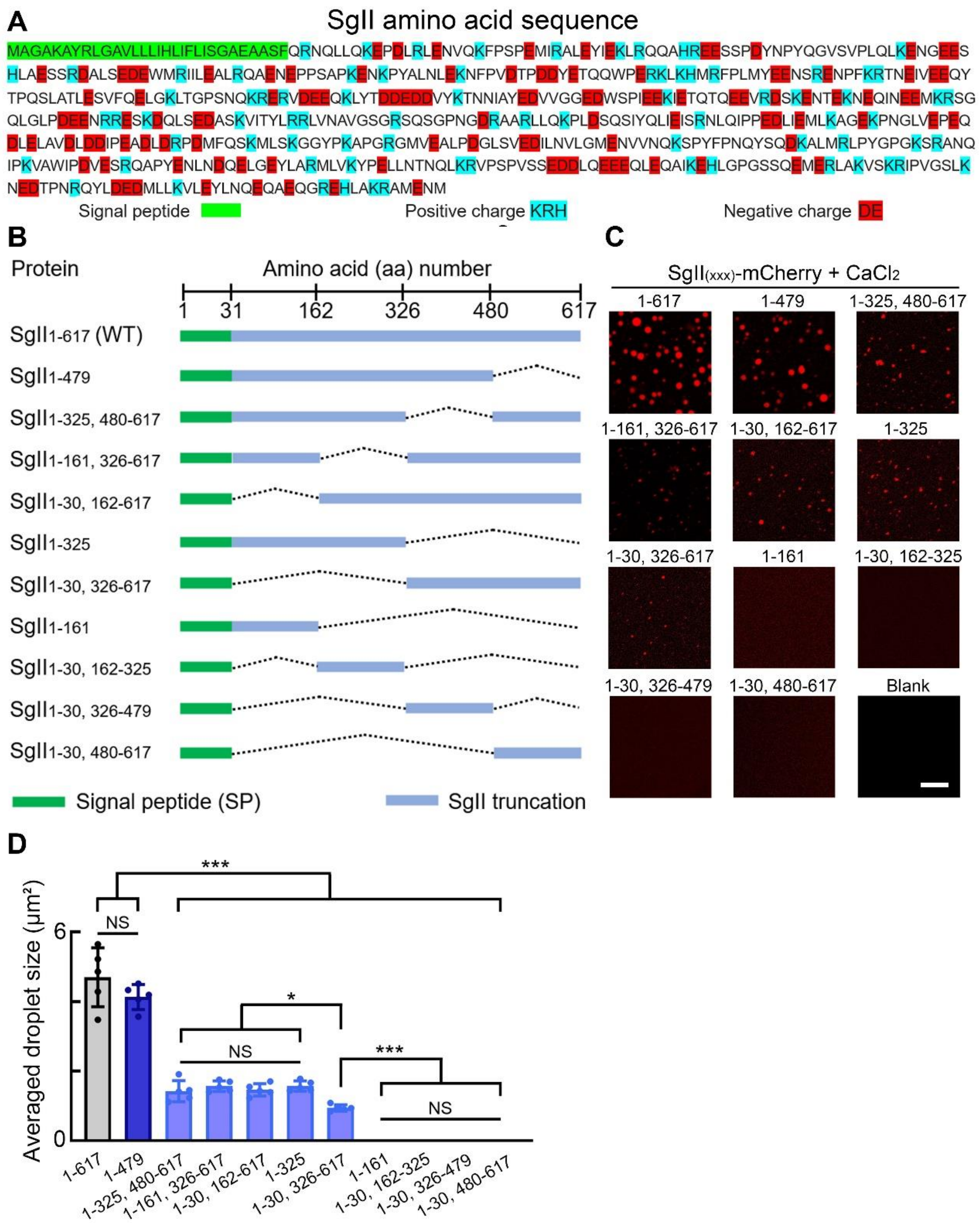

Figure S5 (related to Figures 5A-5D). Different SgII truncations result in various levels of phase separation.

(A) The SgII sequence [green, signal peptide; cyan, positively-charged amino-acids (lysine, K; arginine, R; histidine, H), and red, negatively-charged amino-acids (aspartic acid, D; glutamic acid, E)].

(B) SgII truncation design patterns. Signal peptide (SP) with SgII truncations of different lengths were constructed and used for subsequent experiment.

(C) Typical fluorescence micrographs of mCherry-tagged SgII truncations with 50 mM Ca^{2+} corresponding to panel (B) (scale bar, 20 μm).

(D) Statistics of average phase droplet size of mCherry-tagged SgII truncations corresponding to panel (C) (droplets are from 5 fluorescence micrographs per group, one-way ANOVA). Data are presented as the mean ± SEM with scatter dots. *P < 0.05, ***P < 0.001; NS, no significant difference.

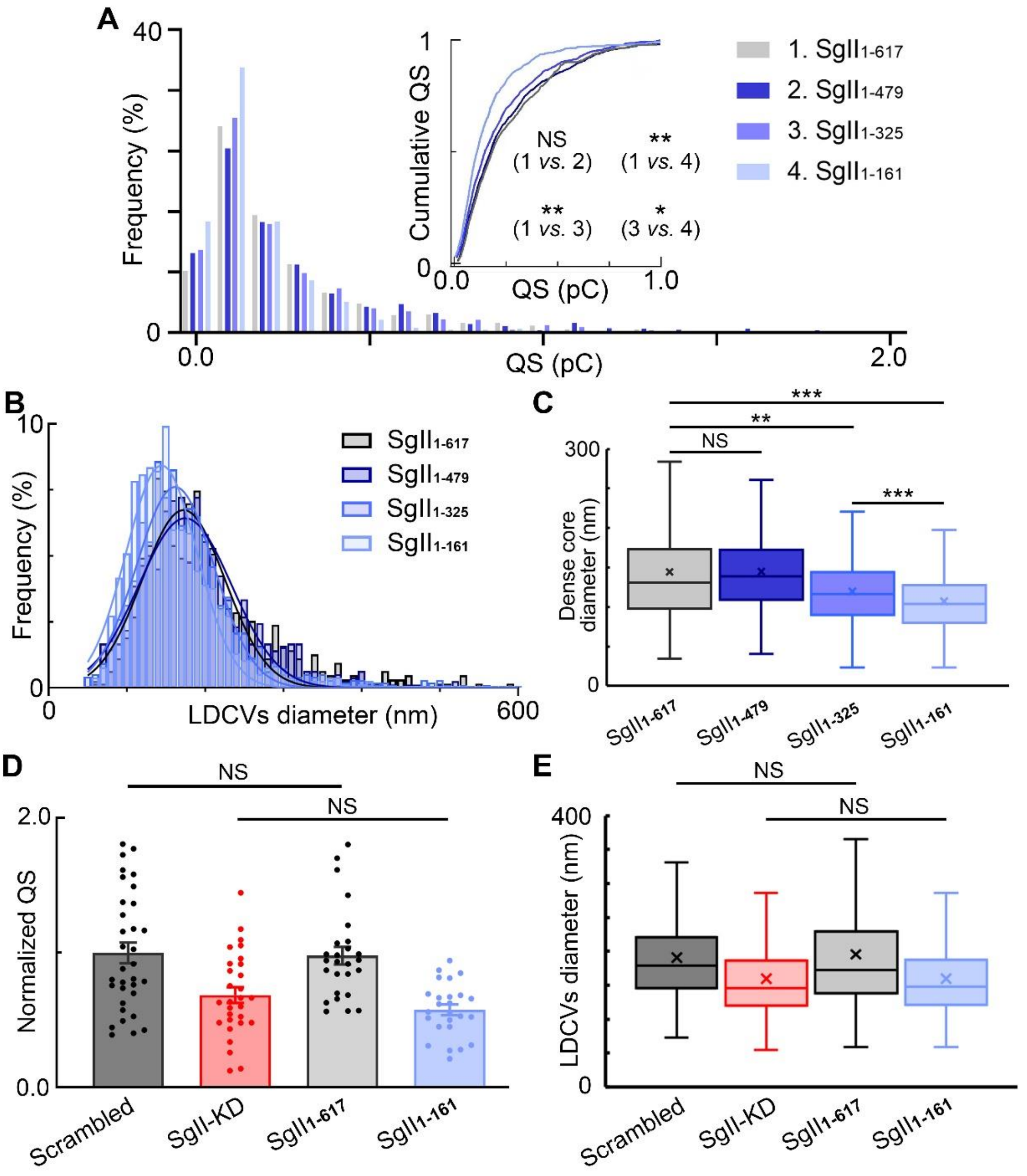

Figure S6 (related to Figures 5E-5H). SgII truncations with different phase separation ability tune the quantal size and vesicle size of LDCVs in ACCs.

(A) Frequency distribution and cumulative curve of QS in SgII truncations rescued ACCs, corresponding to Figure 5F (Kolmogorov-Smirnov test).

(B) Distribution of LDCV diameters in SgII truncations rescued ACCs, corresponding to Figure 5H.

(C) Statistics of dense core diameters corresponding to Figure 5G (Kolmogorov-Smirnov test).

(D) Statistics of normalized quantal size (QS) in scrambled, SgII-KD (shRNA1), $SgII_{1\text{-}617}$ (WT) rescued, and $SgII_{1\text{-}161}$ rescued ACCs, corresponding to Figure 5F (one-

way ANOVA).

(E) Statistic of LDCV diameters in scrambled, SgII-KD (shRNA1), $SgII_{1-617}$ (WT) rescued, and $SgII_{1-161}$ rescued ACCs, corresponding to Figure 5H (Kolmogorov-Smirnov test). Data are presented as the mean ± SEM with scatter dots (D) or the medians with interquartile ranges (C and E). *P < 0.05, **P < 0.01, ***P < 0.001; NS, no significant difference.

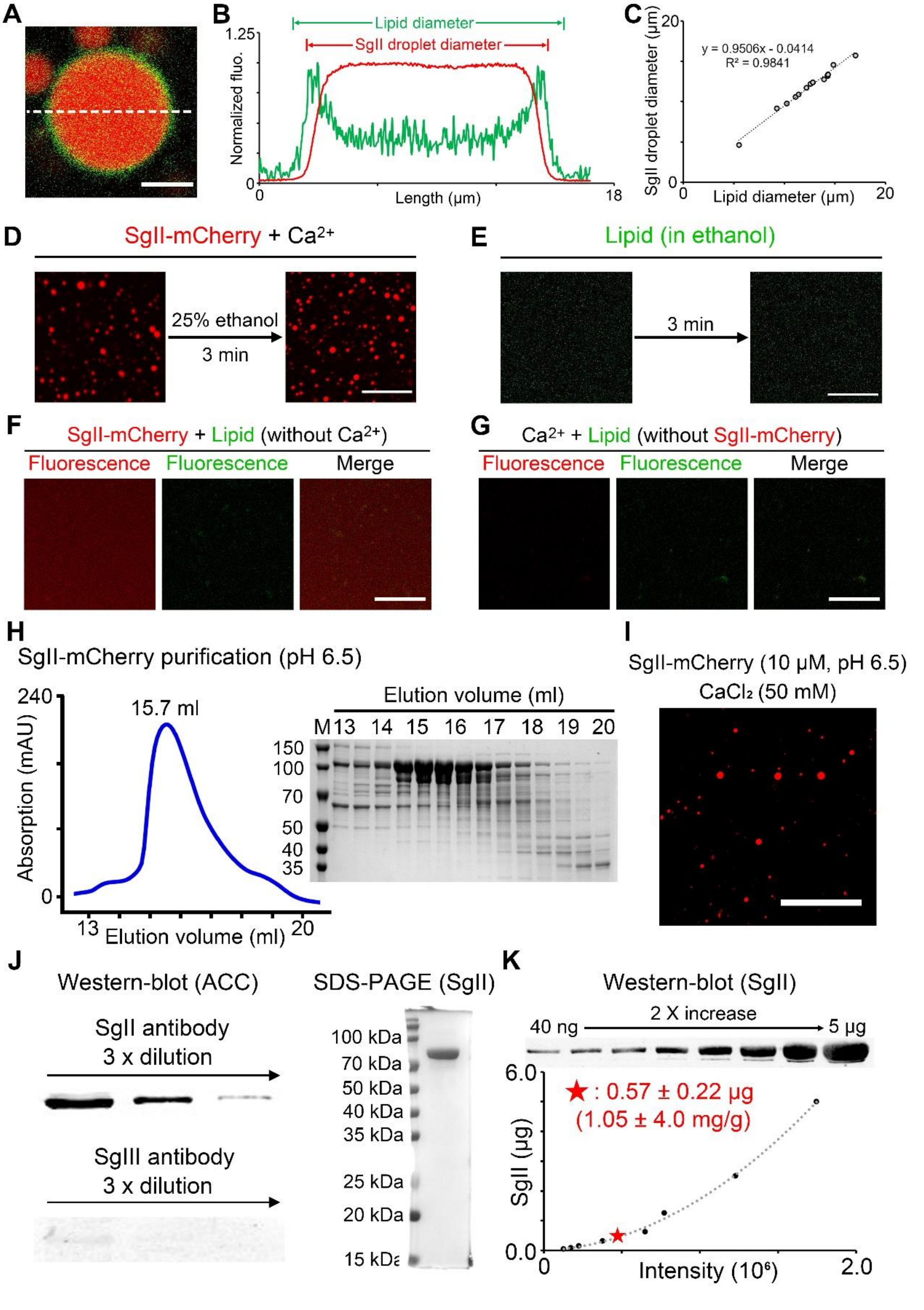

Figure S7 (related to Figures 5I and 5J). SgII phase droplets recruit reconstituted bio-lipids and SgII can form phase droplets at pH 6.5.

(A) Zoom-in view of lipid-coated SgII-mCherry phase droplet. The fluorescent intensity under white line was measured (scale bar, 5 μm).

(B) Plot profiles of normalized fluorescent intensity between SgII-mCherry and lipid under white line in (A). SgII-mCherry droplet and lipid diameters were defined and labelled.

(C) The data dots represent co-incident diameters of coated lipid and SgII-mCherry droplets.

(D) SgII-mCherry phase droplets are not influenced by 25% (v/v) ethanol (scale bar, 20 μm).

(E) Lipids can totally dissolve in ethanol and do not form green circular structures (scale bar, 20 μm).

(F) SgII-mCherry phase droplets are not observed with lipid alone (scale bar, 20 μm).

(G) Circular lipid structures are not observed with Ca^{2+} alone (scale bar, 20 μm).

(H) Left panel, size exclusion chromatography (SEC) profile of SgII purification at pH 6.5 (Na-Citrate). Right panel, SDS-page analysis of SgII peak fractions from SEC result at pH 6.5. Molecular weight markers (kDa) are labeled in lane M.

(I) SgII-mCherry forms phase droplets with 50 mM $CaCl_2$ at pH 6.5 (scale bar, 20 μm).

(J) Left panel, Western blot results of SgII and SgIII in native ACCs from six mice adrenal medullas. The fluorescence intensity decreases gradually along with the dilution. Right panel, SDS-PAGE shows the high purity of *in vitro* standard SgII sample.

(K) Quantitative curve between the weight of SgII purified *in vitro* and its fluorescence intensity by Western blot. The red star shows the averaged SgII amount and the estimated concentration of SgII in native ACCs is 1.05 ± 4.0 mg/g (n = 3 independent experiments).

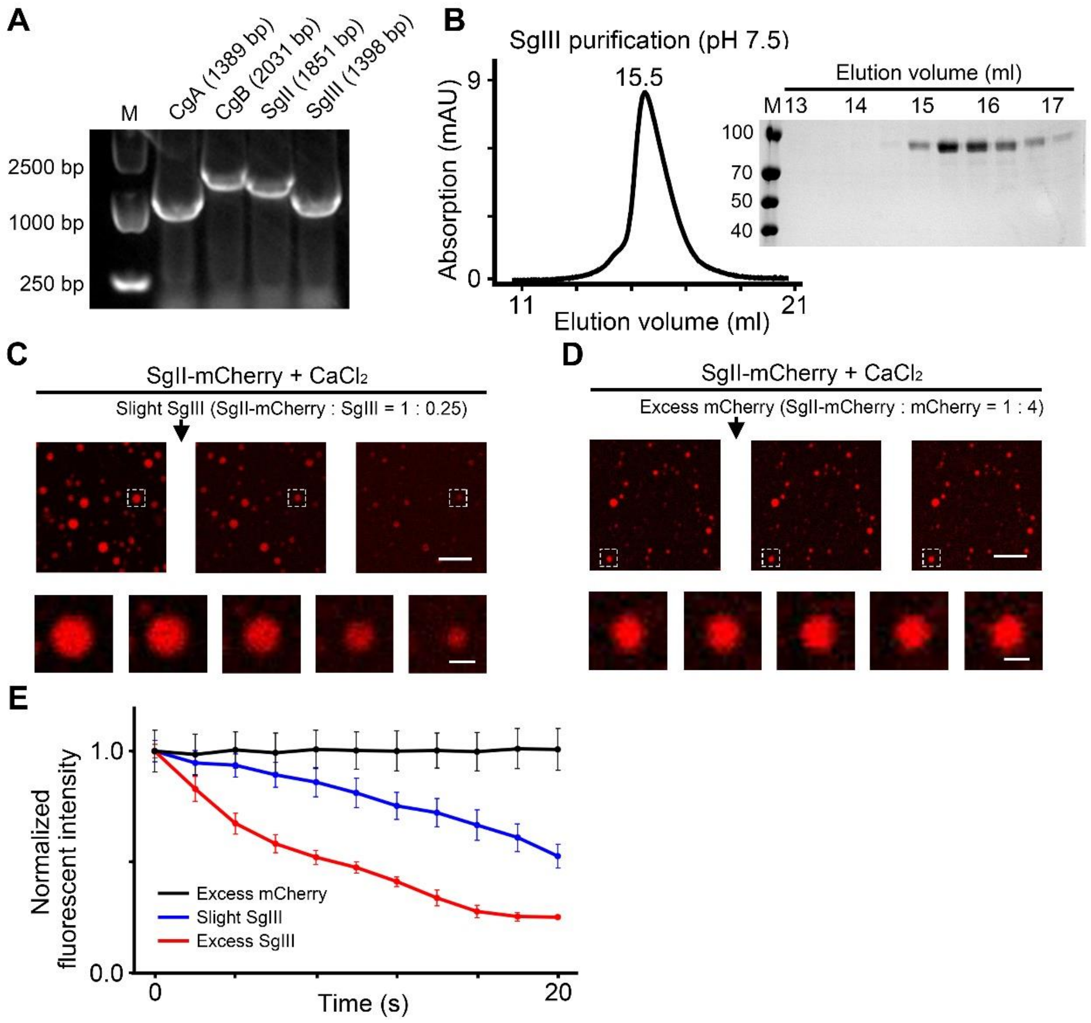

Figure S8 (related to Figures 6A and 6B). SgIII abolishes SgII phase separation.

(A) CgA (1389 bp), CgB (2031 bp), SgII (1851 bp), and SgIII (1398 bp) are successfully amplified from the cDNA of adrenal medulla chromaffin cells by PCR. DNA fragment size markers (bp) are labeled in lane M.

(B) Left panel, size exclusion chromatography (SEC) profile of SgIII purification at pH 7.5. Right panel, SDS-page analysis of SgIII peak fractions from pure SgIII SEC at pH 7.5. Molecular weight markers (kDa) are labeled in lane M.

(C) Compared with Figure 6B, slight SgIII (SgII-mCherry : SgIII (molar ratio) = 1 : 0.25) also diminishes SgII phase droplets but at a slower speed (scale bar, 40 μm).

(D) As a negative control, excess control protein mCherry (SgII-mCherry : mCherry (molar ratio) = 1 : 4) does not affect SgII phase droplets (scale bar, 40 μm).

(E) Fluorescence intensity of SgII droplets incubated with excess SgIII (red), slight SgIII (blue), and excess control protein mCherry (black) (n = 8 droplets per group).

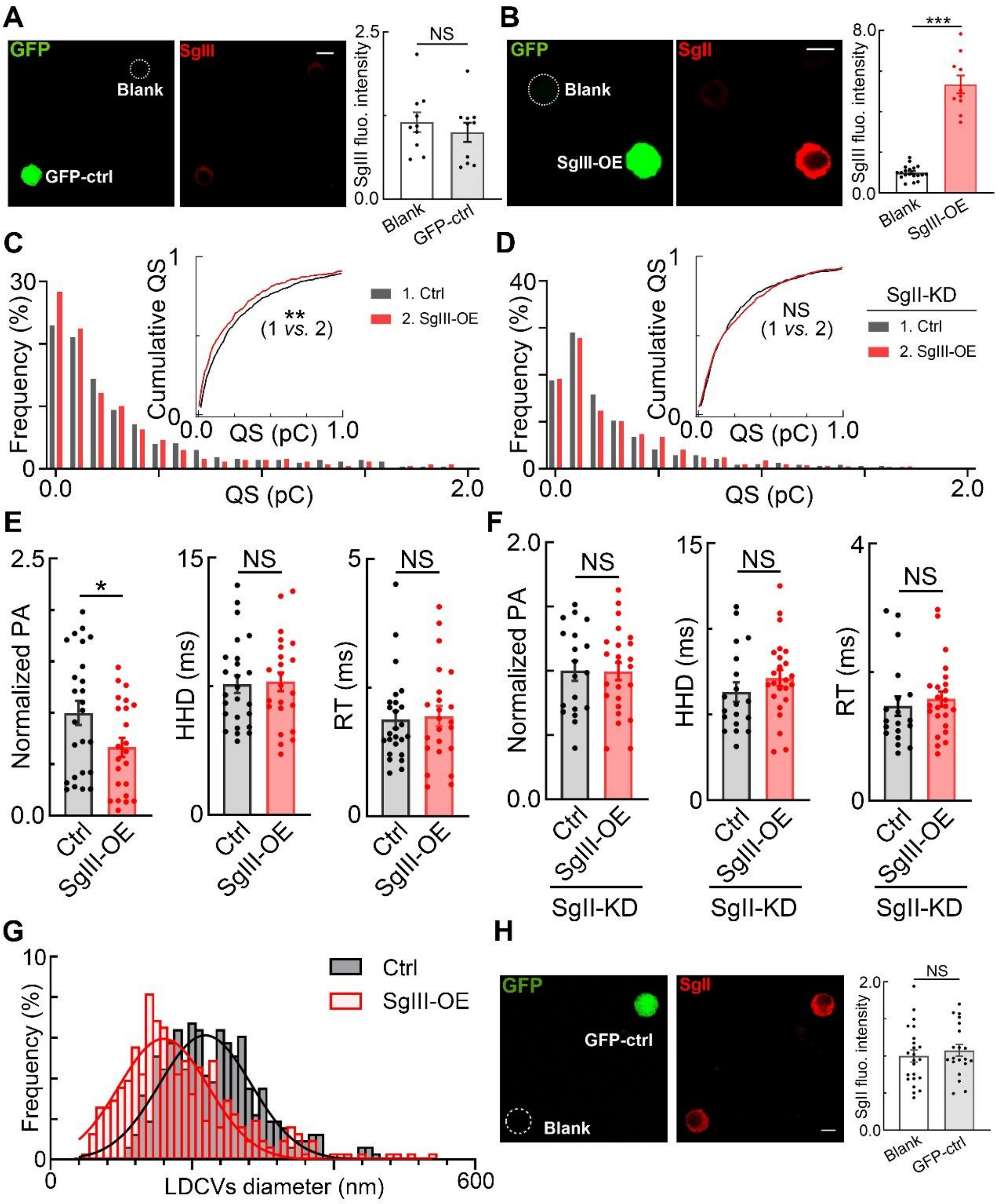

Figure S9 (related to Figures 6C-6J). SgIII-OE reduces quantal catecholamine release *via* SgII in ACCs.

(A and B) For (A), SgIII immunostaining (left) and statistics (right) results in the blank ACCs and the GFP-ctrl plasmid transfected ACCs (scale bar, 10 μm). SgIII expression is not altered by the GFP-ctrl plasmid (n = 10 for blank cells, 10 for GFP-ctrl cells, unpaired Student' *t* test). For (B), SgIII immunostaining (left) and statistics (right) results in the blank ACCs and the SgIII-OE plasmid transfected ACCs (scale bar, 10 μm). SgIII-OE plasmid indeed upregulates the SgIII expression level (n = 19

for blank cells, 12 for SgIII-OE cells; unpaired Student' *t* test).

(C) Frequency distribution and cumulative curve of QS in control and SgIII-OE ACCs, corresponding to Figure 6D (Kolmogorov-Smirnov test).

(D) Same as (C), but in control and SgIII-OE ACCs after SgII-KD, corresponding to Figure 6F (Kolmogorov-Smirnov test).

(E) Statistics of normalized peak amplitude (PA), half-height duration (HHD), and rise time (RT) from amperometric recordings in control and SgIII-OE ACCs (n = 25 for control cells, 23 for SgIII-OE cells; unpaired Student's *t* test).

(F) Statistics of normalized PA, HHD and RT from amperometric recordings of control and SgIII-OE in SgII-KD ACCs (n = 19 for control cells, 24 for SgIII-OE cells; unpaired Student's *t* test). SgIII-OE effect on quantal release is abolished in SgII-KD ACCs.

(G) Distribution of LDCVs diameters in control and SgIII-OE ACCs, corresponding to Figure 6H.

(H) SgII immunostaining (left) and statistics (right) results in the blank ACCs and the GFP-ctrl plasmid transfected ACCs (scale bar, 10 μm). SgII expression is not altered by the GFP-ctrl plasmid (n = 9 for blank cells, 14 for GFP-ctrl cells; unpaired Student's *t* test). Data are presented as the mean ± SEM with scatter dots (A, B, E, F and H). *P < 0.05, **P < 0.01, ***P < 0.001; NS, no significant difference.

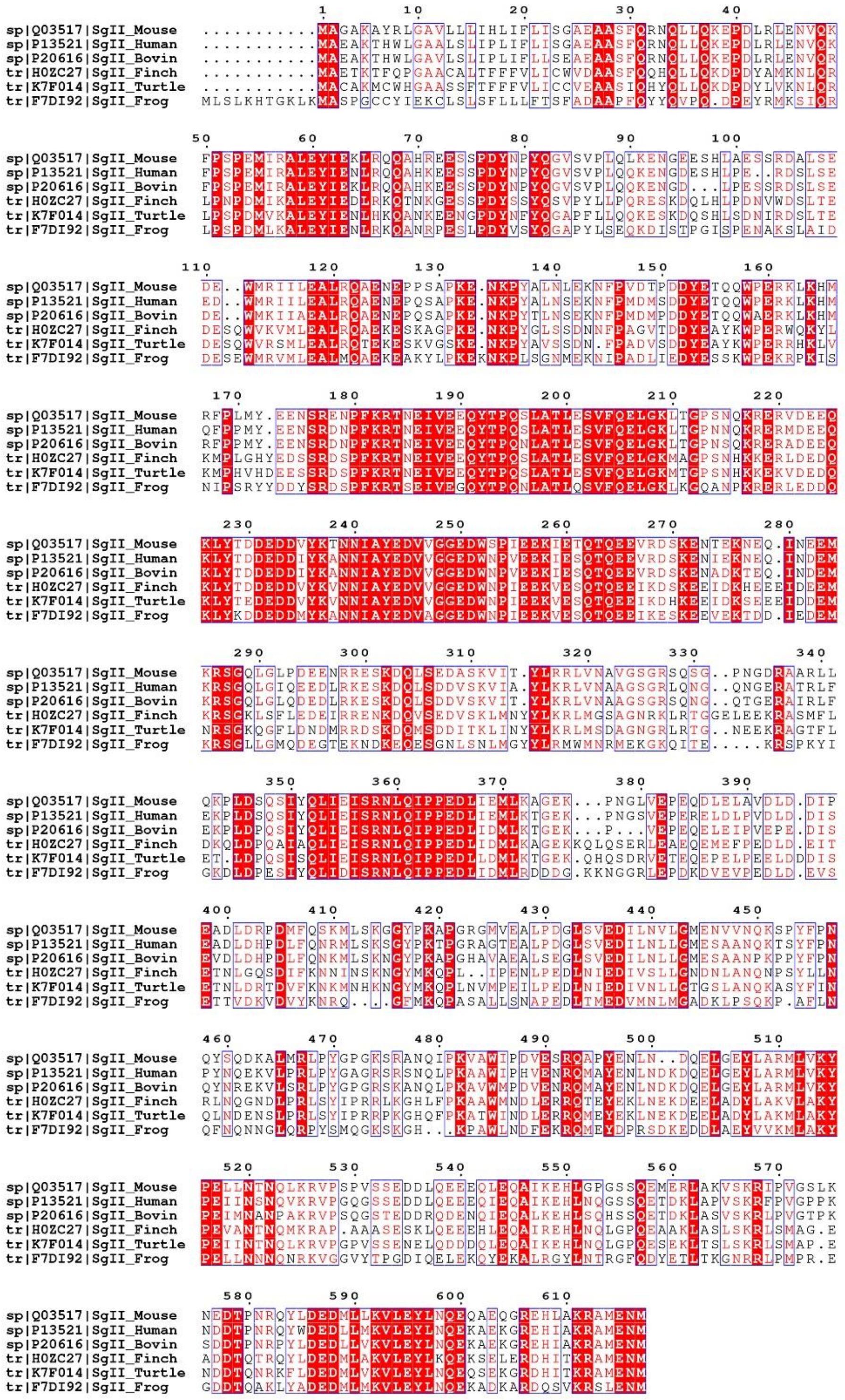

Figure S10. SgII is conserved across species.

Sequence alignment of SgII in six species: *Mus musculus* (mouse, UNIPROT access code Q03517), *Homo sapiens* (human, P13521), *Bos taurus* (bovine, P20616), *Taeniopygia guttata* (finch, H0ZC27), *Pelodiscus sinensis* (turtle, K7F014), and *Xenopus tropicalis* (frog, F7DI92) [2]. The sequence homology of SgII in mouse with

human, bovine, finch, turtle, and frog is 90%, 88%, 74%, 74%, and 69%, respectively.

Movies S1–S7 and video legends:

Movie S1. Live recording of NPY-pHluorin release in scrambled ACCs under TIRF microscopy

Single LDCV release in scrambled ACCs can be visualized by NPY-pHluorin and analyzed (scale bar, 5 μm).

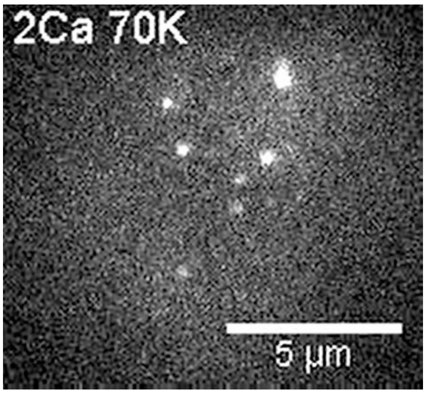

Movie S2. Live recording of NPY-pHluorin release in SgII-KD ACCs under TIRF microscopy

Single LDCV release in SgII-KD ACCs can be visualized by NPY-pHluorin and analyzed (scale bar, 5 μm).

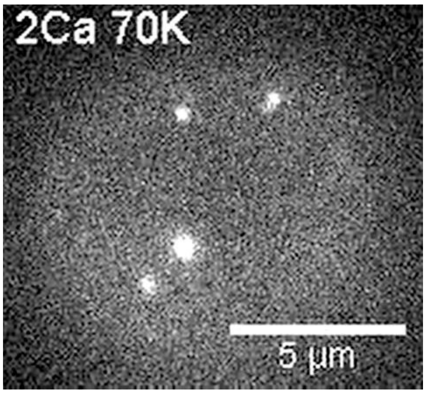

Movie S3. SgII-mCherry droplets can fuse with each other

10 μm SgII-mCherry (pH 7.5) at room temperature forms droplets with 50 mM Ca^{2+}, and the droplets fuse with each other (scale bar, 30 μm).

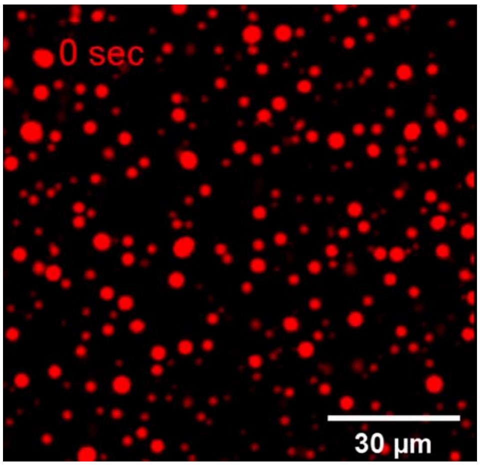

Movie S4. FRAP assay of SgII-mCherry droplets

Photobleaching of two SgII-mCherry droplets, and the recovery of fluorescence. The photobleaching occurs at 20 s (scale bar, 2 µm).

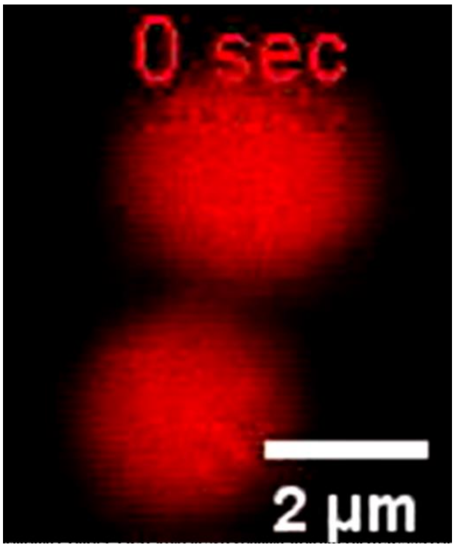

Movie S5. 1,6-hexanediol dissipates SgII-mCherry droplets

5% 1,6-hexanediol is added to SgII-mCherry droplets, and all droplets dissipate in 5 min (scale bar, 30 µm).

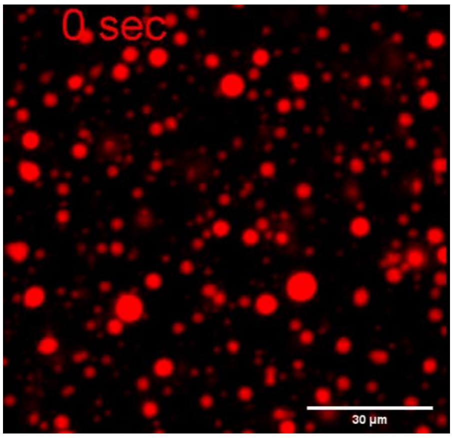

Movie S6. FRAP assay of SgII granular structures in HEK293A cells

Photobleaching of SgII granular structure in HEK293A cells, and the recovery of fluorescence. The photobleaching occurs at 10 s (scale bar, 10 μm).

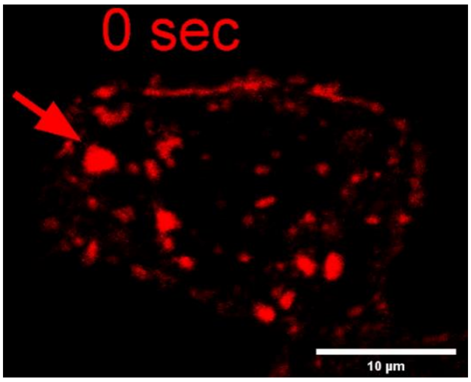

Movie S7. SgIII abolishes SgII-mCherry droplets

Excess SgIII is added to SgII-mCherry droplets, and all droplets immediately dissipate (scale bar, 20 μm).

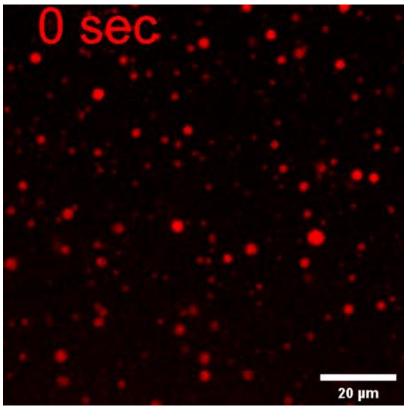

References

[1] Q. Wu, Q. Zhang, B. Liu, Y. Li, X. Wu, S. Kuo, L. Zheng, C. Wang, F. Zhu, Z. Zhou, *J Neurosci.* **2019**, *39*, 199-211.

[2] X. Robert, P. Gouet, *Nucleic Acids Res.* **2014**, *42*, W320-324.
